# Accurate profiling of microbial communities for shotgun metagenomic sequencing with Meteor2

**DOI:** 10.1186/s40168-025-02249-w

**Published:** 2025-11-06

**Authors:** Amine Ghozlane, Florence Thirion, Florian Plaza Oñate, Franck Gauthier, Emmanuelle Le Chatelier, Anita Annamalé, Mathieu Almeida, Stanislav D. Ehrlich, Nicolas Pons

**Affiliations:** 1 Institut Pasteur, Université Paris Cité, Bioinformatics and Biostatistics Hub, F-75015 Paris, France; 2 Université Paris-Saclay, INRAE, MGP, F-78350 Jouy-en-Josas, France; 3 Department of Clinical and Movement Neurosciences, University College London, London, UK

**Keywords:** Shotgun metagenomics, Microbiome, Software, Benchmark, Taxonomic profiling, Functional profiling, Strain profiling

## Abstract

**Background:**

The characterization of complex microbial communities is a critical challenge in microbiome research, as it is essential for understanding the intricate relationships between microorganisms and their environments. Metagenomic profiling has advanced into a multifaceted approach, combining taxonomic, functional, and strain-level profiling (TFSP) of microbial communities. Here, we present Meteor2, a tool that leverages compact, environment-specific microbial gene catalogues to deliver comprehensive TFSP insights from metagenomic samples.

**Results:**

Meteor2 currently supports 10 ecosystems, gathering 63,494,365 microbial genes clustered into 11,653 metagenomic species pangenomes (MSPs). These genes are extensively annotated for KEGG orthology, carbohydrate-active enzymes (CAZymes) and antibiotic-resistant genes (ARGs). In benchmark tests, Meteor2 demonstrated strong performance in TFSP, particularly excelling in detecting low-abundance species.

When applied to shallow-sequenced datasets, Meteor2 improved species detection sensitivity by at least 45% for both human and mouse gut microbiota simulations compared to MetaPhlAn4 or sylph. For functional profiling, Meteor2 improved abundance estimation accuracy by at least 35% compared to HUMAnN3 (based on Bray–Curtis dissimilarity). Additionally, Meteor2 tracked more strain pairs than StrainPhlAn, capturing an additional 9.8% on the human dataset and 19.4% on the mouse dataset.

Furthermore, in its fast configuration, Meteor2 emerges as one of the fastest available tools for profiling, requiring only 2.3 min for taxonomic analysis and 10 min for strain-level analysis against the human microbial gene catalogue when processing 10 M paired reads — operating within a modest 5 GB RAM footprint. We further validated Meteor2 using a published faecal microbiota transplantation (FMT) dataset, demonstrating its ability to deliver an extensive and actionable metagenomic analysis. The unified database design also simplifies the integration of TFSP outputs, making it straightforward for researchers to interpret and compare results.

**Conclusions:**

These results highlight Meteor2 as a robust and versatile tool for advancing microbiome research and applications. As an open-source, easy-to-install, and accurate analysis platform, Meteor2 is highly accessible to researchers, facilitating the exploration of complex microbial ecosystems.

Video Abstract

**Supplementary Information:**

The online version contains supplementary material available at 10.1186/s40168-025-02249-w.

## Background

Metagenomics has transformed the study of microbial communities by enabling comprehensive analysis of genetic material directly from environmental samples. This approach overcomes the limitations of traditional culturing techniques, providing deep insights into the diversity, functional potential, and dynamics of microbial ecosystems. Despite its complexity, integrating taxonomic, functional, and strain-level profiling (TFSP) is crucial for a complete understanding of microbial community structures and their roles in various environments [1–3].

Reference-based profiling has simplified metagenome analysis by alleviating the challenges associated with metagenomic assembly, such as the need for high sequencing depth and the difficulty of assembling genomes from low-abundance microorganisms. Current methodologies typically use genome databases [4–7], species-specific marker genes [8] or universal single-copy marker genes databases [9, 10] for taxonomic profiling of metagenomes. To date, the bioBakery [11] suite is the only all-in-one platform that tackles the challenges of TFSP by incorporating MetaPhlAn4 (taxonomy), HUMAnN3 (functional), and StrainPhlAn (strain). The ChocoPhlAn [8] database serves as the foundation of the bioBakery suite, featuring a collection of species-specific marker genes from diverse environments. However, its adaptability to a wide range of ecosystems (via conserved marker genes) also limits its ability to provide direct insight into functions, as ecosystem-specific functions require secondary annotation workflows. Consequently, comprehensive analysis requires additional tools and pan-genome databases, which can lead to potential discrepancies between the results of different tools.

An alternative strategy involves leveraging microbial gene catalogues tailored to specific microbial ecosystems. This specialization allows one to manipulate smaller, yet highly effective, reference sets that are well-suited for comprehensive taxonomic and functional annotation [12–14]. Evaluating their effectiveness in providing TFSP compared to other methodologies is crucial. Additionally, ensuring that the use of microbial gene catalogues for TFSP is automated and user-friendly is essential for widespread adoption of this approach.

### Our contribution

We present Meteor2, expanding upon the original Meteor [15] tool, which is focused on gene quantification. Meteor2 is engineered to provide TFSP using microbial genes catalogues. It employs Metagenomic Species Pan-genomes [16] (MSP) as its main analytical unit, grouping genes based on co-abundance and designating “signature genes” as the most highly connected and reliable indicators for detecting, quantifying, and characterizing a species. Meteor2 integrates for each catalogue three key functional annotation repertoires: (1) KO (functional orthologs) from the KEGG [17] database, (2) carbohydrate-active enzymes [18] (CAZymes), and (3) antibiotic resistance genes (ARGs), which can be quantified at both the MSP and individual gene levels. In addition to taxonomic and functional profiling, Meteor2 enables strain-level analysis by tracking single nucleotide variants (SNVs) in the signature genes of MSP, providing a more detailed understanding of microbial communities’ dynamics. When computational resources are limited, Meteor2 also provides a “fast mode” for taxonomic and strain profiling that uses a lightweight version of the catalogues containing only signature genes. This option enables rapid and cost-effective analyses while preserving essential profiling features.

## Methods

### Microbial gene catalogues

The Meteor2 database currently includes 10 microbial gene catalogues, covering a variety of environments: human (oral, intestinal, skin), chicken (caecal) and intestinal for dog, cat, rabbit, mouse, pig, and rat. The typical workflow for generating these catalogues from metagenomic data comprises several key steps: read quality trimming, host read removal, metagenomic assembly, gene prediction, gene clustering, gene binning, and gene annotation. In some instances, catalogues incorporate additional data from isolate-derived genomes, as detailed in the methods section of the associated catalogue repositories (see Table S1).

We standardized the annotations across all catalogues. Metagenomic bins were built using MSPminer (v1.1.3), which clustered genes based on co-abundance into Metagenomic Species Pan-genomes (MSPs). Genes exhibiting stable copy numbers across metagenomes, particularly single-copy genes, cluster more readily than those with a variable copy number. Taxonomic annotation was conducted by aligning MSPs against the Genome Taxonomy Database (GTDB r220) using blastn (v2.15.0) with parameters word_size 16. Species-level assignments were made if ≥ 50% of genes matched a representative genome with ≥ 95% mean identity and ≥ 90% mean gene length coverage. Otherwise, MSPs were assigned to a higher taxonomic level (genus to superkingdom) if ≥ 50% of genes shared the same annotation.

Functional annotations were obtained using three complementary approaches. First, we assigned KEGG Orthology (KO) annotations to each gene using the KEGG database with KofamScan (v1.3.0). Second, dbCAN3 [19] (v4.1.4) was employed to annotate carbohydrate-active enzymes (CAZYmes), using default parameters. For ARG, we provided three different annotations of the catalogues. We used Resfinder [20] (v4.6.0) with ResFinderDB (v2.4.0) to identify clinically relevant ARGs from culturable pathogens. To extend coverage beyond clinical isolates, we complemented this with blastn [21] (v2.16.0) against ResfinderFG [22] v2.0 for genes captured by functional metagenomics, applying criteria of 90% identity and 80% gene length coverage. Finally, we used PCM [23] to predict genes associated with 20 families of antibiotic resistance genes, using default parameters, providing genes with structural mechanistic insights.

Meteor2 identifies three types of functional modules: Gut Brain Modules [24] (GBMs), Gut Metabolic Modules [25] (GMMs) and KEGG modules. GBMs are composed of modules annotated with KO, eggNOG [26], or TIGRFAM [27]. These modules are identified by searching catalogue annotations against TIGRFAM 15.0 using hmmsearch (v3.2.1) with the –cut_ga and –noali options, and against eggNOG 3.0 using DIAMOND [28] (v0.9.22) with blastp –sensitive –evalue 1e-5 –min-score 60 –max-target-seqs 100 options. In contrast, GMMs and KEGG modules are uniquely composed of modules derived from KO annotations.

Additionally, a subset catalogue of 100 signature genes per MSPs (the most connected genes among co-abundant genes in the MSP) was created for all catalogues, enabling rapid taxonomical and strain-level profiling of metagenomic samples (i.e, Meteor2 “fast” mode). However, because reducing the catalogue size can increase the risks of misalignments and false positives, the default parameters for gene and species detection are more stringent than in the full catalogue version.

### Implementation

**Fig. 1:**
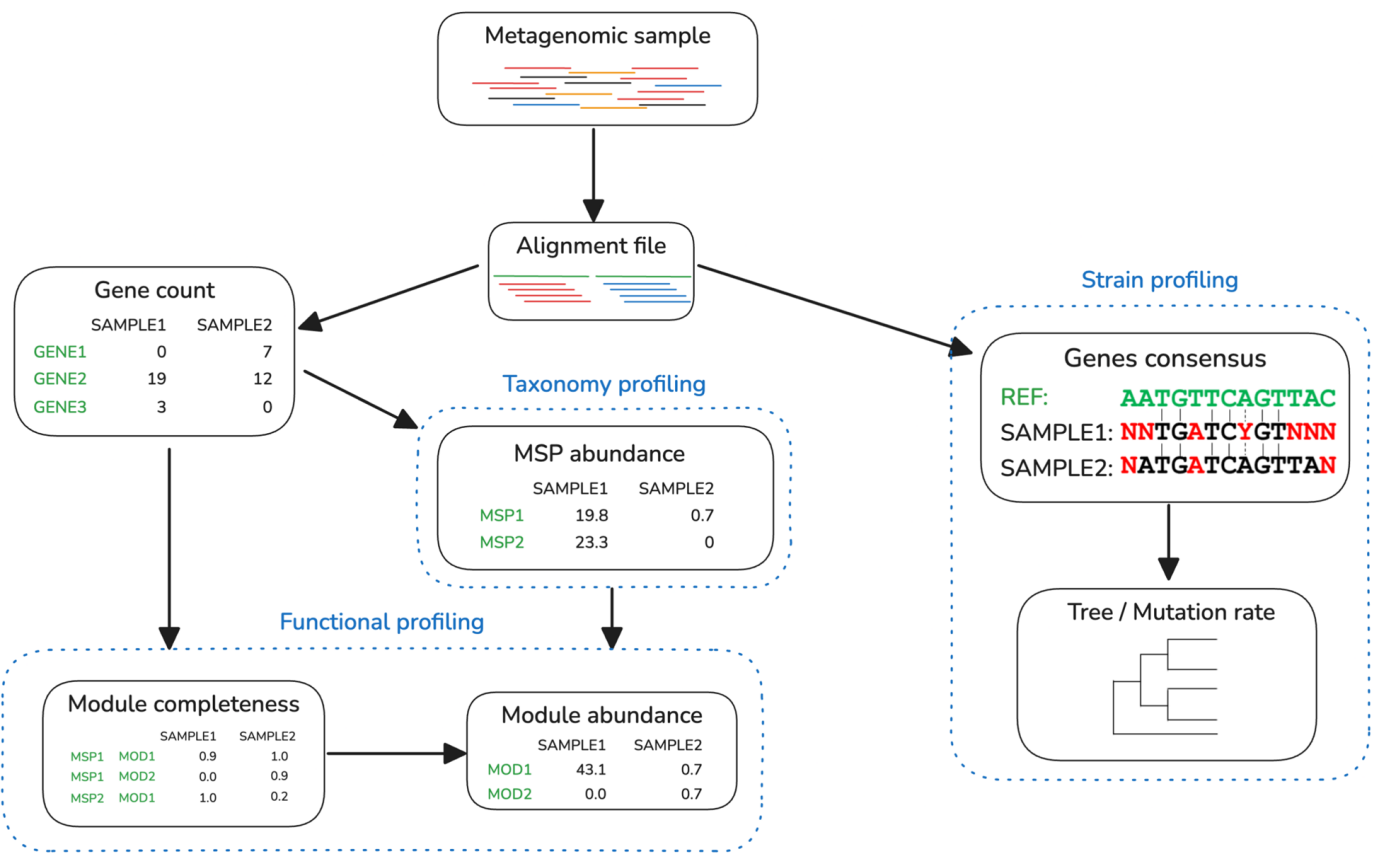
Overview of the Meteor2 pipeline for TFSP analysis. The pipeline proceeds in two main branches, initiating with the alignment of the metagenomic sample data against a comprehensive microbial gene catalogue. The primary branch utilizes this alignment to characterize the microbial community’s taxonomic and functional profile, encompassing abundance of the genes, MSPs and functional modules. The second branch employs SNV calling to construct a consensus gene catalogue, enabling the generation of sample-specific MSPs. These MSPs are then compared to produce phylogenetic analyses, offering a deeper understanding of the microbial community dynamics

Meteor2 is an open-source program that estimates the abundance of genes, species, and functions within microbial communities and tracks strain-level dissemination (Fig. 1). The core of the pipeline involves mapping metagenomic reads against a microbial gene catalogue using bowtie2 [29] (v2.5.4). By default, only alignments of trimmed-to-80nt reads with identity above 95% are considered, though a more stringent threshold of 98% is applied in fast mode.

From these mapping results, Meteor2 estimates gene counts using one of three computation modes: unique, total, or shared counting [23] (default). In total mode, the count of a gene is the sum of all reads aligning to it. Unique mode counts only reads with a single alignment. In shared mode, reads with multiple alignments contribute to the count calculation based on proportion weights. Specifically, a read’s weight is distributed across the n genes it aligns to (where *n* > 1), in proportion to each gene’s unique read count. If all unique counts are zero, the read is distributed evenly (1/n added to each gene's count).

### Taxonomic profiling

Gene count tables are normalized and reduced to generate an MSP profile of samples. Normalization options include depth coverage, defined as the read count divided by gene length and multiplied by 100, with gene length corrected for rate loss as described in Li et al. [30] (default) or FPKM (Fragments Per Kilobase Million). Reduction involves averaging the abundance of signature genes within each MSP after normalization. By default, Meteor2 considers the 100 most central signature genes per MSP. An MSP’s abundance is set to 0 if fewer than 10% of its signature genes are detected. For rapid taxonomic annotations, this threshold is set at 20%.

### Functional profiling

Meteor2 computes the abundance of a specific function by aggregating either the abundances of genes annotated with this function or the abundance of the MSPs that harbor it. When considering MSPs, the analysis is restricted to include only genes that are detected (≥ 1 mapped read) in the sample.

Regarding a functional module, an MSP is considered a module carrier if module completeness (i.e., the proportion of a module’s functional units detected in the sample) is greater than 90%. Since a module step may consist of alternative functions (e.g., K1 and [K2 or K3]), the completeness of a module is calculated for each alternative configuration (e.g., [K1 and K2] on the one hand, [K1 and K3] on the other hand) and the maximum value is retained. The cumulative abundance of these carriers constitutes the module’s potential, which is accompanied by a list of contributing species, specific to a given sample. This approach establishes a robust link between taxonomic and functional profiling, providing a comprehensive understanding of the relationships between microbial community composition and functional capacity.

### Strain-level analysis

Strain level analysis in Meteor2 involves three stages. Firstly, Meteor2 constructs a sample-specific catalogue by performing single nucleotide variant (SNV) calling using Freebayes [31] (v1.3.8), configured for haploid mode without considering INDELs, thereby avoiding the complexities of multiple sequence alignment. Deletions can be difficult to distinguish from depth coverage fluctuations or gene boundary issues. SNVs are identified in pooled continuous mode, requiring a minimum depth coverage of 3 and a minimum non-reference frequency of 0.1. The haplotype length is set to 0, allowing independent evaluation of each potential variant site. Consensus for each position is obtained using the IUPAC [32] codes when multiple SNVs are detected at a given position, and low depth coverage sites are masked with an “*N*” to address positions with depth coverage below 3.

The second stage involves reconstructing sample-specific MSPs with sufficient gene breadth coverage. At least 80 out of 100 signature genes (by default) must have a minimum breadth coverage of 0.5 to be considered adequate for further analysis.

Finally, MSPs that are present in at least 2 samples are considered for mutation rate analysis. Sites with 100% of “N” are disclosed. A minimum of 10 informative sites (default) are required to compute the mutation rate. The distance between each sample-specific MSP is computed pairwise using a General Time Reversible (GTR) evolutionary model. This leverages the inherent alignment of the consensus sequences across samples, as variations consist solely of substitutions without indels. Using Cogent3’s EstimateDistance function, we compute nucleotide substitution rates directly from these pre-aligned sequences to derive the overall mutation rate through GTR model implementation. This method incorporates equilibrium nucleotide frequencies and substitution parameters.

### Benchmark

#### Generation of simulated metagenomic samples

We applied CheckM2 [33] to perform quality control on genomes derived from isolates and Metagenome-Assembled Genomes (MAGs) collected from three sources: the Unified Human Gastrointestinal Genome (UHGG) catalogue [34], the Mouse Gastrointestinal Bacterial Catalogue [35] (MGBC) and the Murine Intestinal Microbiota Integrated Catalog v2 [36] (MIMIC2). We applied a stringent quality selection criterion to these genomes, requiring a completeness of 90% or higher, a contamination rate of less than 5%, and an N50 contig size of at least 10 kbp. We then performed taxonomic annotation of these high-quality genomes using GTDB-Tk [37] with the GTDB R207 release. To minimize potential biases related to database representativeness, we only selected genomes annotated at species-level in GTDB and represented in the databases of the evaluated tools using an in-house reference. Finally, we selected the top four highest-quality genomes per species, resulting in 1920 genomes for the mouse gut microbiome (representing 534 species) and 3,194 genomes for the human gut microbiome (representing 914 species). Details about the number of genomes per species are available in Table S2.

Next, we used wgsim [29] to simulate paired-end short reads from these genomes. The simulation parameters were set to generate 10 M read pairs of 100 bp with an insert size of 350 bp. The human simulated samples were designed to reflect the species abundance profiles from the MetaCardis BMIS project (PRJEB37249), featuring an average of 165 ± 43 species per sample (*n* = 100 samples). For the mouse samples, we followed species abundance profiles from five independent studies (detailed in Table S3), resulting in an average of 160 ± 33 species per sample (*n* = 62 samples). In both cases, each species was represented by a single genome per sample. These datasets constitute the gold standard for human and mouse samples, respectively, and provide a basis for evaluating the performance of the tools being assessed across all three facets of TFSP.

#### Taxonomic profiling benchmark

We evaluated the performance of several taxonomic profiling tools, including Meteor (v2.0.18) in both ‘complete’ and ‘fast’ modes, MetaPhlAn4 (v4.1.1) along with ChocoPhlAn vOct22, mOTUs [9] (v3.0.3), sylph [38] (v0.5.1), SingleM [10] (v0.15.1) with database [39] v3.1.0, Kraken2 (v2.1.3) with Bracken [7] (v2.9), KMCP [6] (v0.9.4), and ganon2 [5] (v2.0.0), on 162 simulated samples. For Meteor2, we used version 8 of the human gut catalogue [40] for human samples and the mouse gut catalogue [36] for mouse samples. The Kraken2 database for GTDB r207 was obtained from Struo2 project (http://ftp.tue.mpg.de/ebio/projects/struo2/GTDB_release207/kraken2/). All tools were run with their default parameters, except for Bracken, which required 200 reads to consider the presence of a species.

To ensure cross-tool comparability, all taxonomic profiles were converted to GTDB r207 and converted to relative abundance. When multiple taxonomical units (i.e. MSPs from Meteor2, species-level genome bins—SGBs—from MetaPhlAn4, or mOTUs) were assigned to the same GTDB species, they were merged into a single unit and their relative abundances were summed. If a tool provided a fraction of unmapped or undetermined metagenome (e.g. SingleM, mOTUs, or MetaPhlAn4), this fraction was removed at that stage. We then applied the OPAL benchmarking framework [41] to compute sensitivity, specificity F1-Score and Bray–Curtis dissimilarity on log10-transformed profiles. However, we did not use OPAL directly (i) to preserve the integrity of unclassified fractions reported by tools like SingleM that OPAL would otherwise renormalize (potentially biasing performance assessment), and (ii) to compute the alpha-diversity index (richness) using original taxonomic units without GTDB species-level compression, thereby avoiding artificial reduction of diversity counts through merging of distinct genomic lineages.

Given that GTDB annotation revealed that a single mOTU could be assigned to different species (e.g., the mOTU ref_mOTU_v3_02367 corresponds to both *Phocaeicola vulgatus* and *Phocaeicola dorei*), we manually curated the taxonomic annotation of mOTUs with multiple GTDB assignments to (i) remove incorrect assignments (e.g., the *Phocaeicola vulgatus/dorei* mOTU ref_mOTU_v3_02367 was also incorrectly assigned to Gastranaerophilaceae CAG-196 sp002102975) and (ii) establish the List of GTDB species from gold standard that could not be distinguished. Notably, we merged gold standard abundance of 4 *Enterobacter* sp. (*E. kobei*, *E. ludwigii*, *E. roggenkampii* and *E. hormaechei A*) into a single species to match mOTUs ref_mOTU_v3_00077. The list of curated species is available in Table S4 and S5.

#### Functional profiling benchmark

To facilitate a comparison between Meteor2 and HUMAnN3, we utilized Uniref90 as the common annotation framework, given its native integration with HUMAnN3. We functionally annotated the MAGs from the gold standard using DIAMOND (v2.1.9) against Uniref90 (version uniref90_201901b_full) with specific parameters: blastp search, 90% identity and 80% gene length coverage, consistent with the settings used in Franzosa et al*.* study [42]. These annotation parameters were also applied to annotate the genes from both human and mouse microbial gene catalogues.

Subsequently, we profiled the simulated samples using Meteor2 (v2.0.18) and HUMAnN3 [11] (v3.8), in conjunction with MetaPhlAn4 (v4.1.1) and the ChocoPhlAn vOct22 database. Since Uniref90 profiles are not inherently part of Meteor2’s outputs, we computed gene-level and species-level Uniref90 abundances using the gene and MSPs profiles, adhering to the standard Meteor2 functional analysis strategy (see Implementation).

HUMAnN3 provided the total abundance of Uniref90 families, as well as each species contribution (including an ‘unclassified’ contribution from direct mapping of reads on Uniref90 database). However, when attempting to map HUMAnN3 species to MetaPhlAn4 SGBs, we encountered limitations due to species that could not be uniquely associated with a single SGB. Using the provided correspondence file from HUMAnN3, we identified species with either no association or multiple associations (e.g. *g__Enterobacter.s__Enterobacter_cloacae*, which was Linked to 18 different SGBs). To maintain the accuracy of our comparisons with the gold standard profile, these species were excluded from further species-level functional analysis, as their SGB assignments were ambiguous or uncertain. 

#### Strain profiling benchmark

We evaluated the performance of strain-level profiling tools, including Meteor2 (v2.0.18), StrainPhlAn4 (ChocoPhlAn vOct22 database), and inStrain [43] (v1.9.0) (MGnify databases [44]: UHGG v2.0, Mouse gut v1.0), using default parameters and our simulated metagenomic samples from the gold standard dataset. Meteor2 was also run in dominant configuration (*f* = 0.5). These tools calculated mutation rates between detected strains using a GTR model, except for inStrain, which calculated ANI. Our gold standard for strain analysis was defined as the ANI between two MAGS of a single species, calculated using skani [45] (v0.2.0) with the “–medium” preset. Intra-species ANI ranged from 95% (species-level) to 100% (near-identical strains) as shown in Fig. S1. To enable comparison with our gold standard, we also aligned inStrain’s databases to the GTDB r207 taxonomy using GTDB-TK (v2.3.2).

### Application case

Reads were retrieved from the NCBI Sequence Read Archive (BioProject ID PRJNA928744) and cleaned using fastp [46] (v0.23.4) with the following parameters: “—cut_front”, “—cut_tail”, “—n_base_limit 0”, “—length_required 60”, “`—trim_poly_g”, “—adapter_sequence CTGTCTCTTATACACATCTCCGAGCCCACGAGAC”, “—adapter_sequence_r2 CTGTCTCTTATACACATCTGACGCTGCCGACGA”. Reads that mapped to the human genome were further discarded using bowtie2 (v2.5.1) against Homo sapiens chm13 t2t v2 reference genome. Samples were profiled using Meteor2 (v2.0.18) (with mapping parameters: normalization = coverage, strain parameters: *f* = 0.5 for dominant configuration and *f* = 0.1 for mixed configuration) and MetaPhlAn4/StrainPhlAn4 (v4.1.1) using ChocoPhlAn vOct22. Additionally, inStrain (v1.9.0) was run with the UHGG database (v2.0).

To investigate the relationship between mutation rates and MAGs, reads were assembled using metaSPAdes [47] (v3.15.5), with contigs shorter than < 1.5 kb filtered. Reads were then aligned to their respective assemblies using strobealign [48]. Binning was performed using COMEBin [49], and quality control of the bins was conducted using CheckM2. Bins with completeness ≥ 70%, contamination ≤ 5%, and N50 ≥ 5 Kb were retained. Taxonomic annotation of the MAGs was performed using GTDB-Tk. Finally, ANI was calculated between all pairs of MAGs assigned to the same species using skani.

### Statistical analysis

All analyses were performed with R4.4.1. To evaluate the relationships between two quantitative variables, Spearman’s correlations were calculated. Comparisons between groups were performed using the Wilcoxon rank-sum test for unpaired data or the Wilcoxon signed-rank test for paired data. The effect size of a quantitative variable between two groups was measured with Cliff’s Delta, as implemented in the effsize package (v0.8.1). Area under the curve (AUC) values were computed using the pROC (v1.1.8.5). Data visualization was performed using a combination of packages, including ggplot2 (v3.5.1), ggpubr (v0.6.0), plotROC (v2.3.1) and ComplexHeatmap (v2.20.0).

## Results

### Meteor2 is a unified platform for high-resolution profiling of species, functions and strains using microbial gene catalogues

The analytical pipeline of Meteor2 comprises four key steps: (i) download of an ecosystem-specific microbial gene catalogue, (ii) alignment of short metagenomic reads to the catalogue, (iii) calculation of abundance profiles of genes, metagenomic species, and functions, (iv) reconstitution of sample-specific MSP genotypes and estimation of their mutation rates (Fig. 1). To achieve accurate and comprehensive results, a non-redundant microbial gene catalogue is an essential prerequisite. Creating such a catalogue requires a balance between comprehensiveness, to ensure good representativeness of the metagenome, and size, to avoid it becoming unwieldy for TFSP.

To address this challenge, Meteor2 relies to this date on 10 curated microbial gene catalogues including human (skin, oral, gut), domestic animals (cat, dog), laboratory models (mice, rat), and livestock (chicken, pig, rabbit) microbiomes. Each catalogue integrates two key components: (i) metagenomic species pangenomes (MSPs), in which genes are clustered by co-abundance, and (ii) unclustered genes which may be missed by metagenome-assembled genomes [50] (MAGs). Meteor2 quantifies species using signature genes that are consistently co-abundant across multiple metagenomic samples. Unlike indirect strategies that search for universal single-copy markers or species-specific core genes through sequence alignment, this data-driven approach directly leverages gene abundance data to identify the most relevant ones for accurate taxonomic profiling. Meanwhile, unclustered genes are also included to capture crucial functions such as virulence factors or antimicrobial resistance; notably, Ruppé et al*.* study [23] found that 13.5% (820 out of 6,095) of predicted antibiotic resistance genes (ARGs) were not clustered within a specific metagenomic species.

In addition, specialized catalogues represent a community-driven effort involving multiple research teams [13, 51–54]. Meteor2 aims to consolidate these resources by selectively integrating them into its profiling repertoire. After being integrated into the Meteor2 workflow, taxonomic and functional annotations are frequently updated to ensure that the catalogues remain up to date. Finally, a key advantage of Meteor2 is its ability to estimate the catalogue representativeness by measuring the proportion of Mapped reads in each sample. This feature provides users with a confidence score regarding both the quality of the generated profiles and the suitability of the selected catalogue for their samples. A Mapping rate of 80–85% is generally considered optimal, accounting for reads arising from intergenic regions or gene boundaries [55].

By integrating this analytical pipeline into a single, streamlined task stratified by ecosystem and utilizing a high-quality microbial gene catalogue, Meteor2 efficiently addresses the TFSP challenge in a unified framework, enabling accurate and comprehensive analysis of microbial communities.

### Meteor2 produces accurate species-level taxonomic profiles

To evaluate the performance of Meteor2 for TFSP, we generated two simulated sequencing reads datasets mimicking, respectively, human (100 samples) or mouse (62 samples) faecal metagenomes using genomes derived from microbial isolates or high-quality MAGs. To ensure that results were independent of database representativity, only species represented in all benchmarked tools were included (see Methods). Using these datasets, we compared the performance of Meteor2 in taxonomic profiling against seven other state-of-the-art tools, including MetaPhlAn4 [8], mOTUs [9], sylph[38], SingleM [10], Kraken2 with Bracken [7], KMCP [6] and ganon2 [5].

**Fig. 2:**
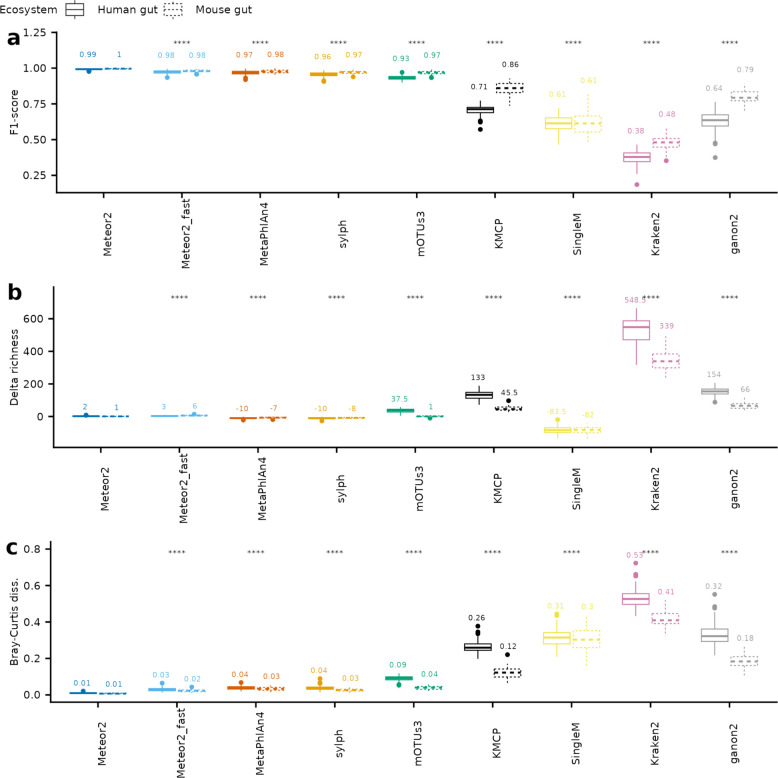
Meteor2 accurately profiles species abundances from synthetic metagenomes. **a** Taxonomic profiling performance according to F1-score (the harmonic mean of the precision and recall of detection) for two synthetic datasets, simulating mouse (62 samples) and human faecal (100 samples) metagenomes, respectively. **b** Richness delta, corresponding to the difference between tools’ estimated richness and gold standard richness. **c** Bray–Curtis dissimilarity values computed between the log10-transformed estimated profiles and the abundances in the gold standard. The median F1-scores, richness deltas and Bray–Curtis dissimilarity values are reported, respectively. *p*-value from Wilcoxon signed-rank tests, **** indicates *p*-value ≤ 0.0001

As anticipated, simulated samples achieved an average read Mapping rate of 84.9% ± 1.9 on both human and mouse gut catalogues. Meteor2 outperformed alternative tools in both species detection and abundance estimation according to the metrics established by the OPAL [41] benchmarking framework. Regarding species detection, Meteor2 demonstrated higher F1-scores (Fig. 2a), indicating a strong balance between sensitivity and specificity (Table S6). Notably, Meteor2 exhibited high specificity, with only two species wrongly detected per sample (median), while MetaPhlAn4 and sylph showed lower sensitivity, failing to detect a median of 9 and 11 species per sample, respectively, often among the low-abundance species (Fig. S2a, b). Consequently, Meteor2 provided the most accurate estimate of species richness (Fig. 2b), outperforming MetaPhlAn4, sylph, and SingleM, which underestimated species richness, while mOTUs3, KMCP, Kraken2, and ganon2 overestimated it.

Regarding species abundance, Bray–Curtis dissimilarity analysis showed that Meteor2’s profiles were the closest to the expected values (Fig. 2c). Notably, Meteor2 accurately estimated the abundance of low-abundance species, while alternative tools exhibited systematic biases, with MetaPhlAn4 underestimating and sylph overestimating their abundances (Fig. S3). These biases became more pronounced as sequencing depth was reduced from 10 to 5 M or 1 M read pairs, with MetaPhlAn4 and sylph presenting significant declines in accuracy, while Meteor2 maintained robust performance (Fig. S4). Furthermore, Meteor2’s “fast mode” provided, with limited computational resources, accurate taxonomic profiles comparable to those of the best tools (Table S7).

### Meteor2 provides direct and specific functional characterization of metagenomic samples

Next, we aimed to compare the accuracy of functional profiles generated by Meteor2 with those produced by HUMAnN3, the most widely used tool. First, we annotated the genes in the genomes used to create the simulated samples with UniRef90 —the HUMAnN3 annotation database. Then, for each simulated sample, we generated gold-standard functional profiles based on the genomes that constituted them and their respective abundance (see Methods). We then applied Meteor2 and HUMAnN3 to the simulated metagenomes and evaluated their performance using two complementary approaches: (1) detection of functional content for species/sample pairs (i.e., Uniref functions detected in a metagenome and attributed to a specific species), and (2) accuracy of the estimated community-level UniRef abundances using the Bray–Curtis dissimilarity.

**Fig. 3:**
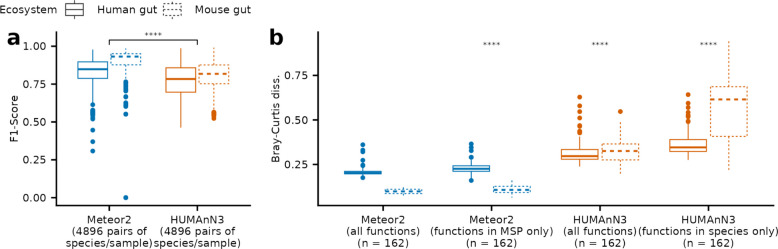
Meteor2 enables precise estimation of functional capacity in synthetic metagenomes. **a** Functional profiling performance according to F1-score for human and mouse synthetic datasets, comparing the accuracy of Meteor2 and HUMAnN3 in detecting functional content at the species/sample. **b** Bray–Curtis dissimilarity values, comparing the estimated functional profiles and the gold standard for both Meteor2 and HUMAnN3 (considering all functions and species contribution signal). *p*-value from Wilcoxon signed-rank tests; **** indicates *p*-value ≤ 0.0001

Our results showed that Meteor2 surpassed HUMAnN3 in functional profiling, achieving a higher F1-score in species/sample functional annotations (Fig. 3a), due to overall superior performance in terms of both sensitivity and specificity (Fig. S5a, b). Of note, Meteor2 retrieved functional information for 99.99% and 99.77% of species/sample pairs with more than 1 × depth coverage for human and mouse metagenomes, respectively (Fig. S5c), whereas HUMAnN3 retrieved only 62.49% and 14.13%, respectively, due to discrepancies between MetaPhlAn4 and HUMAnN3 databases, which hinder the unambiguous mapping of a large part of HUMAnN3 species on MetaPhlAn4 SGBs.

Community-level analysis based on Bray–Curtis dissimilarity further revealed that functional profiles from Meteor2, whether derived from all functions or only those identified in MSPs, were closer to gold standard than those produced by HUMAnN3 (Fig. 3b). This demonstrates Meteor2’s ability to provide robust insights into metagenome functionalities. In practice, Meteor2 can leverage its multi-database support (KEGG, CAZymes, and antibiotic resistance genes; see Methods) to deliver a more comprehensive understanding of metagenomic data.

### Meteor2 enables high-resolution and accurate strain profiling

**Fig. 4:**
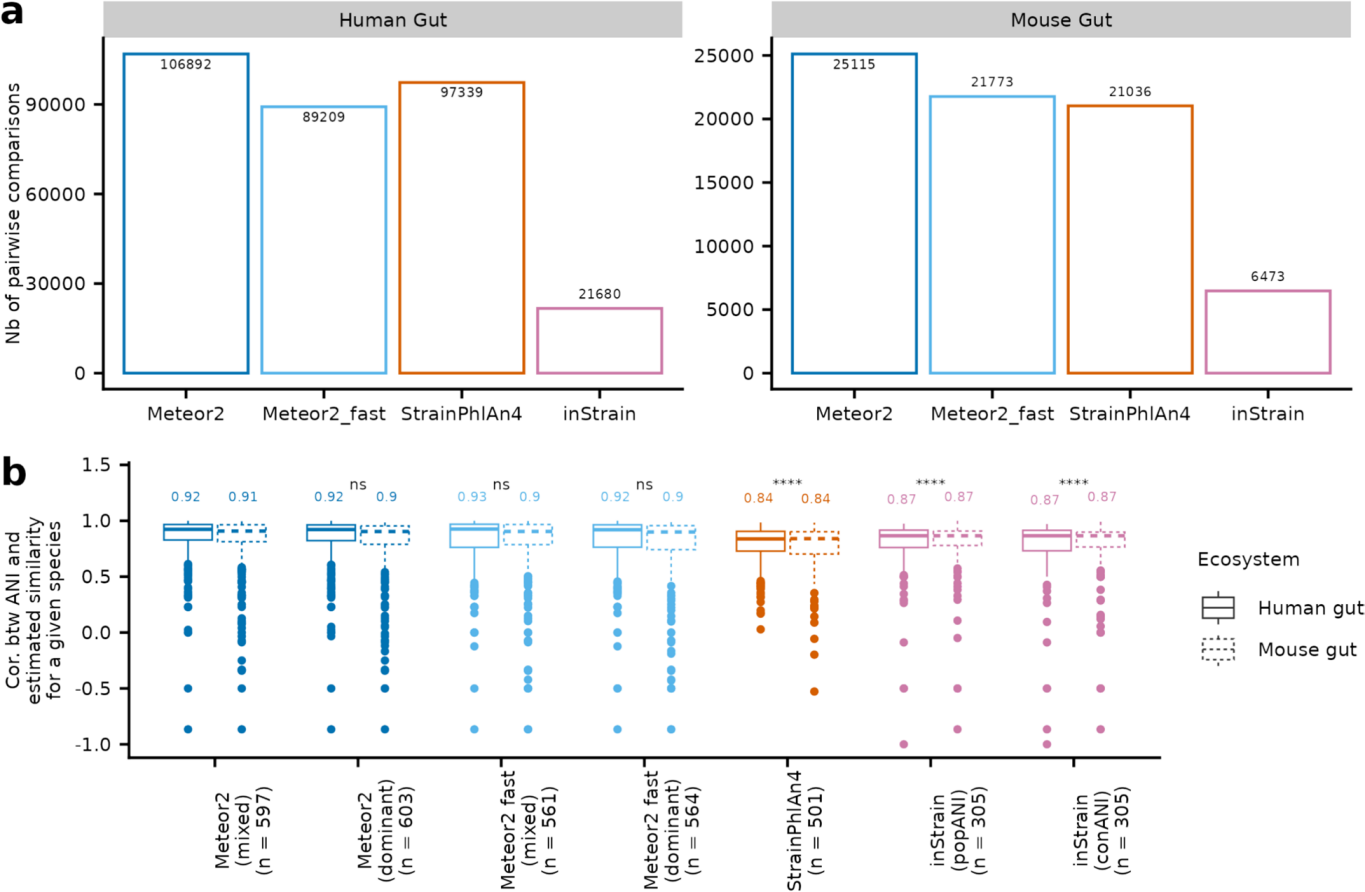
Meteor2 demonstrates superior precision in strain profiling. **a** Number of pairwise comparisons performed per tools. **b** Species-wise Spearman’s correlation coefficient between ANI (gold standard) and similarity estimated by the different tools’ configuration, considering only species with sufficient signal (i.e., a minimum of 3 pairwise comparisons and ANI standard deviation > 0). The analysis included two configurations of Meteor2: “mixed”, which considered all detected alleles at a given position, and “dominant”, which focused solely on the dominant alleles. Similarly, inStrain was evaluated using both conANI (dominant alleles only) and popANI (both dominant and non-dominant alleles). Similarity is assessed either by ANI (inStrain) or by 1—mutation rate (Meteor2, StrainPhlAn4). *p*-value from Wilcoxon tests, ns indicates *p*-value > 0.05, ****, *p*-value ≤ 0.0001

We performed a comparative evaluation of Meteor2 for strain analysis against two state-of-the-art tools: StrainPhlAn4 [8], and inStrain [43]. The three provide a pairwise measure of genetic distance/similarity between strains from distinct metagenomic samples, with Meteor2 and StrainPhlAn4 computing mutation rates and inStrain estimating the average nucleotide identity (ANI). Using our simulated dataset, Meteor2 outperformed StrainPhlAn4 by tracking more strains, thereby yielding a greater number of pairwise mutation rates (Fig. 4a). By contrast, inStrain identified fewer strains, likely because of its stringent depth coverage requirement (5X) for single nucleotide variant (SNVs) detection.

We then compared the estimated genetic distances/similarities between samples with the gold standard ANI (i.e., measured from the genomes used in the simulated metagenomes; see Methods). To align with inStrain dual ANIs reporting per sample pair —conANI for the dominant alleles and popANI for both dominant and non-dominant alleles— Meteor2 was executed in two distinct modes: a “mixed” configuration, where all detected alleles at a given position were considered, and a “dominant” configuration, focusing solely on the dominant alleles (parameter -f). Across both configurations (mixed and dominant) and operating modes (standard and fast), Meteor2 consistently outperformed other tools in terms of species-wise Spearman’s correlation between the estimated similarity and the gold standard ANI (Fig. 4b).

When all species were pooled together, we observed a linear relationship between Meteor2 mutation rates and the true ANI (Fig. S6a). Notably, the commonly used 97% ANI threshold for defining subspecies [56] corresponded to a mutation rate of 0.015. Moreover, in differentiating completely identical MAGs (ANI = 100%) from distinct ones (ANI < 100%), Meteor2 achieved an AUC of 0.99 and 0.94 on human and mouse ecosystems, respectively (Fig. S6b). The optimal cut-off for Meteor2 was 1e-04, at which point its sensitivity was 99% and 98%, and its specificity was 98% and 79% for human and mouse simulated datasets, respectively. Other tools achieved comparable AUC values (Table S8). Meteor2 successfully discriminated strains up to 99.9%, and showed a drop in performance for strains with 99.9% < ANI < 100%, in line with StrainPhlAn4 and inStrain (Fig. S7).

### Meteor2 TFSP delivers detailed insights into the impact of faecal microbiota transplantation

To further validate Meteor2, we applied it to a publicly available metagenomic dataset from a human faecal microbiota transplantation (FMT) study [57], which had been previously processed using the bioBakery suite (MetaPhlAn4, StrainPhlAn4, HUMAnN3). The dataset comprised 71 samples collected from 4 donors and 18 recipients at 4 timepoints: pre-FMT (S1), 1-week post-FMT (S2), 1-month post-FMT (S3), and 3-months post-FMT (S4). The recipients were classified into two groups based on their response to antitumor treatment: responders (R, *n* = 12) and non-responders (NR, *n* = 6). The mean mapping rate achieved with Meteor2, which was 81% ± 3%, indicated that the human gut catalogue provided a representation of the samples close to that of simulated samples.

**Fig. 5:**
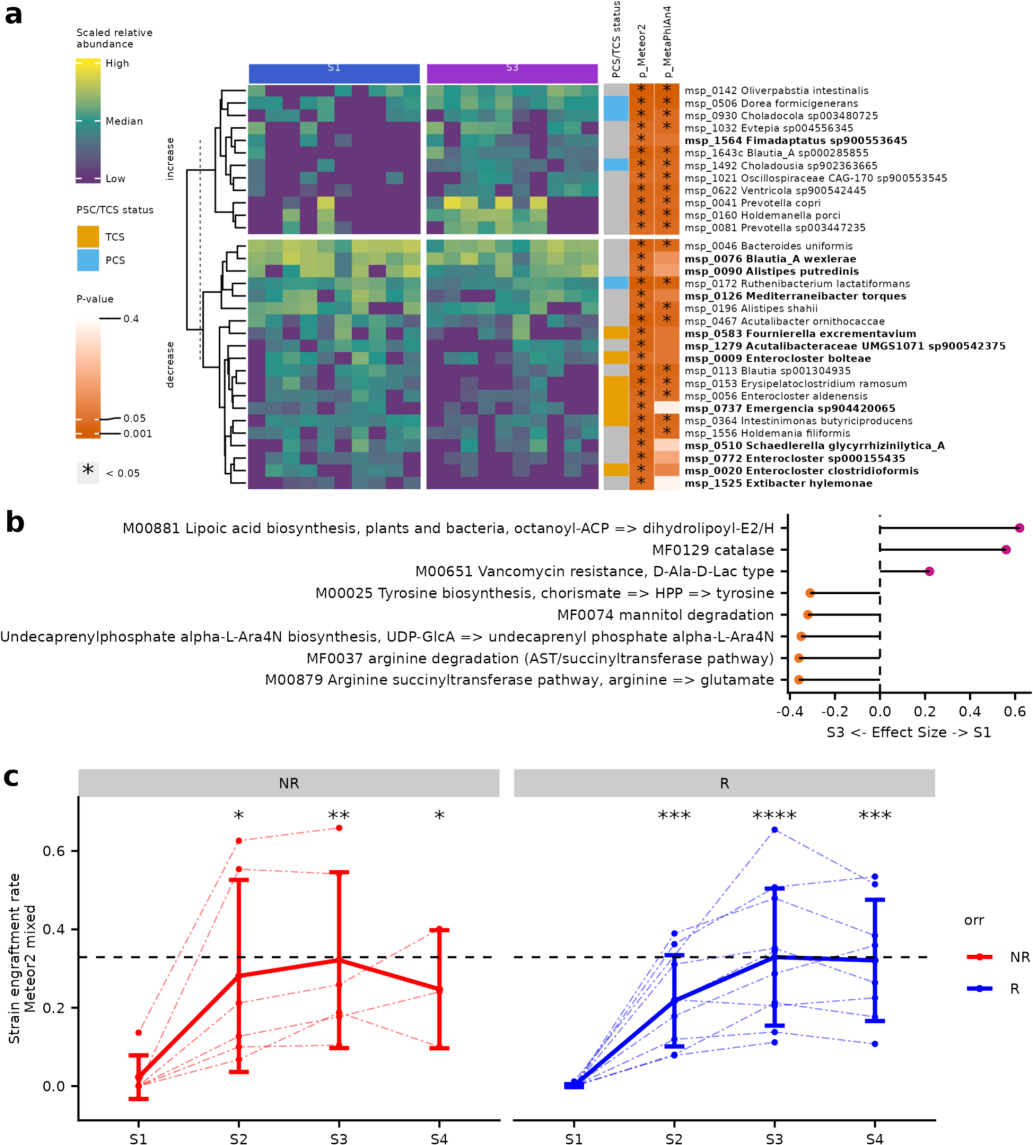
Meteor2 demonstrates efficient TFSP analysis on a real FMT dataset. **a** Differentially abundant MSPs between S1 and S3 in responders only. The bars on the right indicate each species’ status (transient colonizing species (TCS) or persistent colonizing species (PCS), the *p*-values obtained with Meteor2 and MetaPhlAn4 (Wilcoxon signed-rank tests). The correspondence between MSPs from Meteor2 and SGBs from MetaPhlAn4 is based on GTDB r207 annotation. Top panel gathers species that increase between S1 and S3 while bottom panel gathers species that decrease between S1 and S3. Species that are identified as significantly different by Meteor2 alone are highlighted in bold, whereas those detected by both tools are not. **b** Cliff’s Delta effect size for functional modules contrasting S1 and S3 (in responders only, *p*-value from Wilcoxon signed-rank tests). **c** Engraftment rate computed for non-responders (NR) and responders (R) at all timepoints. Each point represents an individual sample; dashed lines connect samples from the same individual. The solid line indicates the mean engraftment rate across all samples at each timepoint, with error bars representing the standard deviation. *p*-values from Wilcoxon tests comparing each timepoint to S1 are shown. * indicates 0.01 < *p*-value ≤ 0.05, **: 0.001 < *p*-value ≤ 0.01, ***: 0.0001 < *p*-value ≤ 0.001, ****: *p*-value ≤ 0.0001

Using Meteor2, our results were highly consistent with those of Routy et al*.* study [57]: alpha-diversity increased significantly from S1 to S4, all patient groups considered (Fig. S8a). Focusing on the S1-S3 contrast of the responder group only (pre-FMT vs post-FMT), we identified 39% more differentially abundant MSPs than species-level genome bins (SGBs) provided by MetaPhlAn4 (*n* = 32 and 23, respectively, Wilcoxon signed-rank test, *p* ≤ 0.05). Moreover, MSPs had lower *p*-values than their SGB counterparts, due to the higher sensitivity of Meteor2 compared to MetaPhlAn4 (Fig. S8b). We also observed a decrease in *Enterocloster* spp. (*E. clostridoformis*, *E. aldenensis*, *E. bolteae* and *E.* sp000155435) from S1 to S3, a bacterial genus known to be associated with gut microbiome dysbiosis (Fig. 5A). Interestingly, four species found significantly depleted at S3 only with Meteor2 profiles are transient colonizing species (TCS), which are microbial species associated with human intestinal dysbiosis [12]. In parallel, we observed almost the same species significantly enriched in S3 compared to SGBs (only *Fimadaptatus* spp. newly detected by Meteor2), among which three persistent colonizing species (PCS), which are species associated with healthy populations. Functional analysis revealed that S1 samples were enriched in catalase and lipoic acid synthesis (Fig. 5b), suggesting that the S1 microbiota contributed to higher oxidative stress and inflammation in the gut environment compared to S3. Given the increased vancomycin resistance (KEGG module) at S1, we investigated the antibiotic resistance profiles of individuals by computing the ratio between genes with resistance (according to PCM [23], ResFinder [20] or ResFinderFG v2.0 [22]) and the total number of genes detected in a sample. We found a significant decrease in ResFinder genes between S1 and S3 (Fig. S8c).

Next, we reconstituted 2,902 MaGs from all samples, enabling the estimation of ANI for approximately 22,000 conspecific genome pairs (see Methods). In addition, we performed strain profiling using Meteor2 (both mixed and dominant configuration), StrainPhlAn, and inStrain, and compared the genetic distance/similarities to the ANI of the corresponding genomes. Since reconstructed MAGs typically represent only the dominant strain of a species in a sample, Meteor2 in dominant configuration (considering only the major alleles) was the tool that inferred genetic distances that correlated most with ANI estimated from MAGs among all profilers and outperformed Meteor2’s mixed configuration (Fig. S8d).

We then investigated the engraftment rate, defined as the fraction of identical strains among all pairs of conspecific strains identified in both a recipient and the donor at a given timepoint. We used Meteor2 in mixed configuration to account for strain mixtures following FMT. Based on benchmarking with simulated data, we applied a mutation rate cut-off of 1e-04 to classify strains as identical. In line with the study design, the rate of shared strains pre-FMT (timepoint S1) was close to 0 (0.1% ± 0.4 and 2.3% ± 5.6 in R and NR, respectively) (Fig. 5c). Consistent with Routy et al*.* study, an increase in engraftment rate was observed in R compared to NR at timepoints S3 and S4, though not at S2. Notably, Meteor2 (mixed), StrainPhlAn, and inStrain (popANI) produced similar engraftment profiles, whereas Meteor2 (dominant) and inStrain (conANI) reported lower engraftment rates at all timepoints (Fig. S9), as both focused on major alleles, reflecting only dominant strains.

Finally, to further highlight Meteor2 capabilities, we integrated both functional and strain-level results to gain deeper insight into *Bacteroides stercoris,* a species for which donors harbor distinct strains and provide sufficient depth coverage for strain analysis, having been detected in *n* = 45 samples. Using complete Linkage clustering and a mutation rate cutoff of 5e-03 —corresponding to 99% ANI— we identified two main clusters (*n* = 17 and *n* = 14): one linked to donor D2 and its corresponding post-FMT samples, and another associated with donor D5 and its post-FMT samples (Fig. S10). By contrast, pre-FMT samples and a subset of post-FMT samples (frequently at timepoint S2) formed isolated clusters or remained as singletons. Notably, a single pre-FMT sample (individual L802-P2) clustered with the D2 cluster, while its associated post-FMT samples (from D5) clustered with the D5 cluster (S3) or were close to clustering with it (S2, S4), highlighting a strain shift. To develop a more comprehensive perspective, we integrated strain clustering information with functional module detection, accessory gene presence/absence, and gene annotations using KEGG, CAZymes, and ARG databases. This analysis showed that the D2 cluster was characterized by a depletion of genes involved in glutamine degradation to glutamate, glutamate biosynthesis, and lysine biosynthesis, whereas the D5 cluster was marked by a depletion of genes involved in histidine biosynthesis. Such depletions in accessory genes supporting these key amino acid pathways could suggest partial auxotrophies [58], requiring *B. stercoris* to rely on alternative metabolic routes or external sources—potentially provided by other community members—to meet its nitrogen requirements [59] and support protein synthesis. This holistic view of strain-level variations, combined with taxonomic and functional profiling, is facilitated by Meteor2’s streamlined use of microbial catalogues.

## Discussion

Meteor2 is a new, integrated, gene-catalogue-based solution for TFSP microbial analysis. Through benchmark with simulated metagenomic samples, we have demonstrated that Meteor2 is a robust profiler for various host-associated microbial ecosystems even at low sequencing depth. This feature is particularly valuable for samples with a high proportion of host DNA (e.g., salivary or skin samples), and where obtaining a high number of microbial reads at a reasonable cost is challenging [60]. More specifically, Meteor2’s high sensitivity enables improved detection and quantification of low-abundance species, which may critically influence host phenotypes [61, 62]. Furthermore, the “fast mode” option in Meteor2 was found to be the second-best performing approach in terms of accuracy for both taxonomy and strain profiling, surpassed only by the standard Meteor2 workflow, while outcompeting other tools in terms of speed and resource efficiency.

Despite these advantages, Meteor2 —and similar approaches— currently focuses on prokaryotes and eukaryotes, underscoring the need to develop methods that incorporate viral sequence data. One promising solution involves integrating the Unified Human Gut Virome (UHGV) catalogue and optimizing analytical parameters for virome analysis. Moreover, the existing ecosystem-specific approach of Meteor2 limits its capacity to trace strain transmission across multiple ecosystems.

Meanwhile, ongoing initiatives, including Le French Gut project [63] and the Israeli gut [64], as well as earlier efforts like Milieu Intérieur [65], Twins UK [66], Swedish [67], Estonian [68] and American gut [69] projects, are now enabling the development of country- and population-specific catalogues. This refinement process could enhance and diversify the resources for robust metagenomic profiling. In parallel, multiple teams worldwide are creating specialized catalogues that span diverse environments and host niches. Following a careful selection process, adhering to the recommendations outlined by Commichaux et al*.* study [70], we plan to integrate and organize these specialized catalogues within Meteor2, thereby expanding its applicability and utility across a broader range of microbiomes.

## Conclusions

Meteor2 unifies taxonomic, functional, and strain-level profiling within a single streamlined pipeline, making it a powerful, high-resolution solution for microbiome research. By leveraging comprehensive and regularly updated microbial gene catalogues, it delivers accurate species-level taxonomic profiles, reliable functional annotations, and robust strain analyses—outperforming or matching state-of-the-art approaches in both simulated and real-world datasets. Overall, Meteor2’s versatile framework, combined with its user-friendly design, enables precise and efficient analysis of complex microbial communities and promises to accelerate future discoveries in microbiome science.

## Supplementary Information

Additional file 1: Table S1. List of gene catalogues currently available through Meteor2. Table S2. Summary of number of genomes used per species to construct the synthetic samples. Table S3. List of Bioprojects whose samples were used to construct the synthetic samples from the mouse gut ecosystem. Table S4. List of GTDB species to merge into a single species for mOTU gold standard. Table S5. List of species (GTDB r207 definition) removed from specific mOTUs taxonomical annotation. Table S6. Metrics of presence/absence calculated on the synthetic datasets. Table S7. Performance in terms of time and memory usage of the different tools on the synthetic datasets. Table S8. AUC of tools’ ability to discriminate between identical genomes (100% ANI) and distinct genomes (< 100% ANI). Figure S1. Pairwise ANI of genomes used to generate simulated samples. Figure S2. Distribution of the depth coverage of true positive and false negative species for MetaPhlAn4 and sylph. Figure S3. Meteor2 accurately profiles high- and low-abundance species from synthetic metagenomes. Figure S4. Taxonomic profiling performance of Meteor2 in comparison to MetaPhlAn4 and sylph for low sequencing depths. Figure S5. Functional profiling performance of Meteor2 in comparison to HUMAnN3. Figure S6. Relationship between pairwise ANI and mutation rate distributions. Figure S7. Strain discrimination performance for genomes from different subspecies. Figure S8. Meteor2 provides insights into a real FMT dataset at diversity, taxonomic, functional and strain level. Figure S9. Engraftment rate computed on real FMT dataset using ANI or mutation rate provided by different tools and different configurations. Figure S10. TFSP integration: clinical, taxonomic, functional and strain information on *Bacteroides stercoris* (msp_0032). Supplementary Information. Le French Gut Consortium Members.

## Data Availability

FMT data are available on the NCBI Sequence Read Archive, BioProject ID PRJNA928744. Simulated metagenomics reads are available here: (10.57745/D3TOEZ) (https:/doi.org/10.57745/D3TOEZ).

## Abbreviations

ANI: Average Nucleotide Identity
ARG: Antibiotic resistant genes
AUC: Area under the curve
CAZYmes: Carbohydrate-active enzymes
FMT: Faecal microbiota transplantation
FPKM: Fragment per kilobase million
GBM: Gut brain modules
GMM: Gut metabolic modules
GTDB: Genome Taxonomy Database
GTR: General Time Reversible
KO: Kegg Orthology
MAG: Metagenome-assembled genome
MGBC: Mouse Gastrointestinal Bacterial Catalogue
MIMIC2: Murine Intestinal Microbiota Integrated Catalog v2
MSP: Metagenomic species pangenomes
NR: Non-responder
PCS: Persistent colonizing species
R: Responder
SGB: Species-level genome bins
SNV: Single nucleotide variant
TCS: Transient colonizing species
TFSP: Taxonomic, functional, and strain-level profiling
UHGG: Unified Human Gastrointestinal Genome
UHGV: Unified Human Gut Virome
