# Supplementary material for: Accurate profiling of microbial communities for shotgun metagenomic sequencing with Meteor2
Source: Microbiome. 2025 Nov 6;13:227. doi: 10.1186/s40168-025-02249-w (PMC12590682; doi:10.1186/s40168-025-02249-w)
Supplement: Supplementary file 2 — Additional file 1: Table S1. List of gene catalogues currently available through Meteor2. Table S2. Summary of number of genomes used per species to construct the synthetic samples. Table S3. List of Bioprojects whose samples were used to construct the synthetic samples from the mouse gut ecosystem. Table S4. List of GTDB species to merge into a single species for mOTU gold standard. Table S5. List of species (GTDB r207 definition) removed from specific mOTUs taxonomical annotation. Table S6. Metrics of presence/absence calculated on the synthetic datasets. Table S7. Performance in terms of time and memory usage of the different tools on the synthetic datasets. Table S8. AUC of tools’ ability to discriminate between identical genomes (100% ANI) and distinct genomes (< 100% ANI). Figure S1. Pairwise ANI of genomes used to generate simulated samples. Figure S2. Distribution of the depth coverage of true positive and false negative species for MetaPhlAn4 and sylph. Figure S3. Meteor2 accurately profiles high- and low-abundance species from synthetic metagenomes. Figure S4. Taxonomic profiling performance of Meteor2 in comparison to MetaPhlAn4 and sylph for low sequencing depths. Figure S5. Functional profiling performance of Meteor2 in comparison to HUMAnN3. Figure S6. Relationship between pairwise ANI and mutation rate distributions. Figure S7. Strain discrimination performance for genomes from different subspecies. Figure S8. Meteor2 provides insights into a real FMT dataset at diversity, taxonomic, functional and strain level. Figure S9. Engraftment rate computed on real FMT dataset using ANI or mutation rate provided by different tools and different configurations. Figure S10. TFSP integration: clinical, taxonomic, functional and strain information on Bacteroides stercoris (msp_0032). Supplementary Information. Le French Gut Consortium Members. [file 40168_2025_2249_MOESM1_ESM.docx]

Supplementary Tables

**Table S1**: List of gene catalogues currently available through Meteor2.

| **Microbial gene catalogue** | **Name** | **Genes count (M)** | **Complete genes (%)** | **Metagenomic Species  Pan-genomes (MSPs)** | **Size (full) (GB)** | **Size (light) (GB)** | **Description** |
| --- | --- | --- | --- | --- | --- | --- | --- |
| *Canis lupus familiaris* | dog_gut | 0.95 | 100.00 | 234 | 1.4 | 0.1 | <https://zenodo.org/records/16982647> |
| *Felis catus* | cat_gut | 1.3 | 100.00 | 344 | 2.0 | 0.2 | <https://zenodo.org/records/16982752> |
| *Gallus gallus domesticus* | chicken_caecal | 13.6 | 100.00 | 2420 | 19.6 | 1.2 | <https://zenodo.org/records/16982807> |
| *Homo sapiens gut* | human_gut | 10.4 | 57.61 | 1990 | 12.6 | 0.7 | <https://zenodo.org/records/16982835> |
| *Homo sapiens oral* | human_oral | 8.4 | 79.75 | 853 | 13.7 | 0.5 | <https://zenodo.org/records/16983006> |
| *Homo sapiens skin* | human_skin | 2.9 | 100.00 | 392 | 3.9 | 0.2 | <https://zenodo.org/records/16982908> |
| *Mus musculus* | mouse_gut | 5.0 | 100.00 | 1252 | 10.3 | 0.6 | <https://zenodo.org/records/16983064> |
| *Oryctolagus cuniculus* | rabbit_gut | 5.7 | 91.14 | 1053 | 8.0 | 0.4 | <https://zenodo.org/records/16983124> |
| *Rattus norvegicus* | rat_gut | 5.9 | 60.40 | 1627 | 7.0 | 0.6 | <https://zenodo.org/records/16983154> |
| *Sus domesticus* | pig_gut | 9.3 | 80.73 | 1523 | 11.3 | 0.7 | <https://zenodo.org/records/16983194> |

**Table S2**: Summary of number of genomes used per species to construct the synthetic samples.

| **Ecosystem** | **Genome count** | **Species count** |
| --- | --- | --- |
| **Human gut** | 1 | 170 |
| **Human gut** | 2 | 121 |
| **Human gut** | 3 | 106 |
| **Human gut** | 4 | 349 |
| **Mouse gut** | 1 | 60 |
| **Mouse gut** | 2 | 50 |
| **Mouse gut** | 3 | 66 |
| **Mouse gut** | 4 | 317 |

**Table S3**: List of Bioprojects whose samples were used to construct the synthetic samples from the mouse gut ecosystem.

| bioproject | # samples | ref |
| --- | --- | --- |
| PRJEB7759 | 25 | <https://doi.org/10.1038/nbt.3353> |
| CNP0000619 | 7 | <https://doi.org/10.1128/msphere.01119-20> |
| PRJNA783624 | 7 | <https://doi.org/10.1158/2159-8290.CD-21-0808> |
| PRJEB40719 | 14 | <https://doi.org/10.1073/pnas.2219431120> |
| PRJNA397886 | 9 | <https://doi.org/10.1128/msystems.00036-17> |
| total | 62 |  |

**Table S4**: List of GTDB species to merge into a single species for mOTU gold standard.

| MOTU | motu_definition | GTDB.R207 | new_gtdb |
| --- | --- | --- | --- |
| ref_mOTU_v3_03695 | Methanobrevibacter smithii [ref_mOTU_v3_03695] | d__Archaea;p__Methanobacteriota;c__Methanobacteria;o__Methanobacteriales;f__Methanobacteriaceae;g__Methanobrevibacter_A;s__Methanobrevibacter_A smithii | d__Archaea;p__Methanobacteriota;c__Methanobacteria;o__Methanobacteriales;f__Methanobacteriaceae;g__Methanobrevibacter_A;s__Methanobrevibacter_A smithii |
| ref_mOTU_v3_03695 | Methanobrevibacter smithii [ref_mOTU_v3_03695] | d__Archaea;p__Methanobacteriota;c__Methanobacteria;o__Methanobacteriales;f__Methanobacteriaceae;g__Methanobrevibacter_A;s__Methanobrevibacter_A smithii_A | d__Archaea;p__Methanobacteriota;c__Methanobacteria;o__Methanobacteriales;f__Methanobacteriaceae;g__Methanobrevibacter_A;s__Methanobrevibacter_A smithii |
| ref_mOTU_v3_03622 | Clostridium sp. [ref_mOTU_v3_03622] | d__Bacteria;p__Firmicutes_A;c__Clostridia;o__Lachnospirales;f__Lachnospiraceae;g__Copromonas;s__Copromonas sp900066535 | d__Bacteria;p__Firmicutes_A;c__Clostridia;o__Lachnospirales;f__Lachnospiraceae;g__Copromonas;s__Copromonas sp900066535 |
| ref_mOTU_v3_03622 | Clostridium sp. [ref_mOTU_v3_03622] | d__Bacteria;p__Firmicutes_A;c__Clostridia;o__Lachnospirales;f__Lachnospiraceae;g__Copromonas;s__Copromonas sp900066055 | d__Bacteria;p__Firmicutes_A;c__Clostridia;o__Lachnospirales;f__Lachnospiraceae;g__Copromonas;s__Copromonas sp900066535 |
| ref_mOTU_v3_03475 | Bacteroides sp. [ref_mOTU_v3_03475] | d__Bacteria;p__Bacteroidota;c__Bacteroidia;o__Bacteroidales;f__Bacteroidaceae;g__Bacteroides;s__Bacteroides ovatus | d__Bacteria;p__Bacteroidota;c__Bacteroidia;o__Bacteroidales;f__Bacteroidaceae;g__Bacteroides;s__Bacteroides ovatus |
| ref_mOTU_v3_03475 | Bacteroides sp. [ref_mOTU_v3_03475] | d__Bacteria;p__Bacteroidota;c__Bacteroidia;o__Bacteroidales;f__Bacteroidaceae;g__Bacteroides;s__Bacteroides xylanisolvens | d__Bacteria;p__Bacteroidota;c__Bacteroidia;o__Bacteroidales;f__Bacteroidaceae;g__Bacteroides;s__Bacteroides ovatus |
| ref_mOTU_v3_03442 | [Clostridium] clostridioforme/bolteae [ref_mOTU_v3_03442] | d__Bacteria;p__Firmicutes_A;c__Clostridia;o__Lachnospirales;f__Lachnospiraceae;g__Enterocloster;s__Enterocloster clostridioformis | d__Bacteria;p__Firmicutes_A;c__Clostridia;o__Lachnospirales;f__Lachnospiraceae;g__Enterocloster;s__Enterocloster clostridioformis |
| ref_mOTU_v3_03442 | [Clostridium] clostridioforme/bolteae [ref_mOTU_v3_03442] | d__Bacteria;p__Firmicutes_A;c__Clostridia;o__Lachnospirales;f__Lachnospiraceae;g__Enterocloster;s__Enterocloster bolteae | d__Bacteria;p__Firmicutes_A;c__Clostridia;o__Lachnospirales;f__Lachnospiraceae;g__Enterocloster;s__Enterocloster clostridioformis |
| ref_mOTU_v3_03437 | [Clostridium] scindens [ref_mOTU_v3_03437] | d__Bacteria;p__Firmicutes_A;c__Clostridia;o__Lachnospirales;f__Lachnospiraceae;g__Clostridium_AP;s__Clostridium_AP sp000509125 | d__Bacteria;p__Firmicutes_A;c__Clostridia;o__Lachnospirales;f__Lachnospiraceae;g__Clostridium_AP;s__Clostridium_AP sp000509125 |
| ref_mOTU_v3_03437 | [Clostridium] scindens [ref_mOTU_v3_03437] | d__Bacteria;p__Firmicutes_A;c__Clostridia;o__Lachnospirales;f__Lachnospiraceae;g__Clostridium_AP;s__Clostridium_AP scindens | d__Bacteria;p__Firmicutes_A;c__Clostridia;o__Lachnospirales;f__Lachnospiraceae;g__Clostridium_AP;s__Clostridium_AP sp000509125 |
| ref_mOTU_v3_03342 | Blautia massiliensis [ref_mOTU_v3_03342] | d__Bacteria;p__Firmicutes_A;c__Clostridia;o__Lachnospirales;f__Lachnospiraceae;g__Blautia_A;s__Blautia_A massiliensis | d__Bacteria;p__Firmicutes_A;c__Clostridia;o__Lachnospirales;f__Lachnospiraceae;g__Blautia_A;s__Blautia_A massiliensis |
| ref_mOTU_v3_03342 | Blautia massiliensis [ref_mOTU_v3_03342] | d__Bacteria;p__Firmicutes_A;c__Clostridia;o__Lachnospirales;f__Lachnospiraceae;g__Blautia_A;s__Blautia_A sp900066205 | d__Bacteria;p__Firmicutes_A;c__Clostridia;o__Lachnospirales;f__Lachnospiraceae;g__Blautia_A;s__Blautia_A massiliensis |
| ref_mOTU_v3_03342 | Blautia massiliensis [ref_mOTU_v3_03342] | d__Bacteria;p__Firmicutes_A;c__Clostridia;o__Lachnospirales;f__Lachnospiraceae;g__Blautia_A;s__Blautia_A sp900066335 | d__Bacteria;p__Firmicutes_A;c__Clostridia;o__Lachnospirales;f__Lachnospiraceae;g__Blautia_A;s__Blautia_A massiliensis |
| ref_mOTU_v3_02367 | Bacteroides dorei/vulgatus [ref_mOTU_v3_02367] | d__Bacteria;p__Bacteroidota;c__Bacteroidia;o__Bacteroidales;f__Bacteroidaceae;g__Phocaeicola;s__Phocaeicola vulgatus | d__Bacteria;p__Bacteroidota;c__Bacteroidia;o__Bacteroidales;f__Bacteroidaceae;g__Phocaeicola;s__Phocaeicola vulgatus |
| ref_mOTU_v3_02367 | Bacteroides dorei/vulgatus [ref_mOTU_v3_02367] | d__Bacteria;p__Bacteroidota;c__Bacteroidia;o__Bacteroidales;f__Bacteroidaceae;g__Phocaeicola;s__Phocaeicola dorei | d__Bacteria;p__Bacteroidota;c__Bacteroidia;o__Bacteroidales;f__Bacteroidaceae;g__Phocaeicola;s__Phocaeicola vulgatus |
| ref_mOTU_v3_02190 | Anaerotignum lactatifermentans [ref_mOTU_v3_02190] | d__Bacteria;p__Firmicutes_A;c__Clostridia;o__Lachnospirales;f__Anaerotignaceae;g__Anaerotignum;s__Anaerotignum sp001304995 | d__Bacteria;p__Firmicutes_A;c__Clostridia;o__Lachnospirales;f__Anaerotignaceae;g__Anaerotignum;s__Anaerotignum sp001304995 |
| ref_mOTU_v3_02190 | Anaerotignum lactatifermentans [ref_mOTU_v3_02190] | d__Bacteria;p__Firmicutes_A;c__Clostridia;o__Lachnospirales;f__Anaerotignaceae;g__Anaerotignum;s__Anaerotignum lactatifermentans | d__Bacteria;p__Firmicutes_A;c__Clostridia;o__Lachnospirales;f__Anaerotignaceae;g__Anaerotignum;s__Anaerotignum sp001304995 |
| ref_mOTU_v3_01657 | Bacteroides thetaiotaomicron [ref_mOTU_v3_01657] | d__Bacteria;p__Bacteroidota;c__Bacteroidia;o__Bacteroidales;f__Bacteroidaceae;g__Bacteroides;s__Bacteroides thetaiotaomicron | d__Bacteria;p__Bacteroidota;c__Bacteroidia;o__Bacteroidales;f__Bacteroidaceae;g__Bacteroides;s__Bacteroides thetaiotaomicron |
| ref_mOTU_v3_01657 | Bacteroides thetaiotaomicron [ref_mOTU_v3_01657] | d__Bacteria;p__Bacteroidota;c__Bacteroidia;o__Bacteroidales;f__Bacteroidaceae;g__Bacteroides;s__Bacteroides faecis | d__Bacteria;p__Bacteroidota;c__Bacteroidia;o__Bacteroidales;f__Bacteroidaceae;g__Bacteroides;s__Bacteroides thetaiotaomicron |
| ref_mOTU_v3_01350 | Streptococcus salivarius [ref_mOTU_v3_01350] | d__Bacteria;p__Firmicutes;c__Bacilli;o__Lactobacillales;f__Streptococcaceae;g__Streptococcus;s__Streptococcus salivarius | d__Bacteria;p__Firmicutes;c__Bacilli;o__Lactobacillales;f__Streptococcaceae;g__Streptococcus;s__Streptococcus salivarius |
| ref_mOTU_v3_01350 | Streptococcus salivarius [ref_mOTU_v3_01350] | d__Bacteria;p__Firmicutes;c__Bacilli;o__Lactobacillales;f__Streptococcaceae;g__Streptococcus;s__Streptococcus vestibularis | d__Bacteria;p__Firmicutes;c__Bacilli;o__Lactobacillales;f__Streptococcaceae;g__Streptococcus;s__Streptococcus salivarius |
| ref_mOTU_v3_00856 | Anaerostipes hadrus [ref_mOTU_v3_00856] | d__Bacteria;p__Firmicutes_A;c__Clostridia;o__Lachnospirales;f__Lachnospiraceae;g__Anaerostipes;s__Anaerostipes sp900066705 | d__Bacteria;p__Firmicutes_A;c__Clostridia;o__Lachnospirales;f__Lachnospiraceae;g__Anaerostipes;s__Anaerostipes sp900066705 |
| ref_mOTU_v3_00856 | Anaerostipes hadrus [ref_mOTU_v3_00856] | d__Bacteria;p__Firmicutes_A;c__Clostridia;o__Lachnospirales;f__Lachnospiraceae;g__Anaerostipes;s__Anaerostipes hadrus_A | d__Bacteria;p__Firmicutes_A;c__Clostridia;o__Lachnospirales;f__Lachnospiraceae;g__Anaerostipes;s__Anaerostipes sp900066705 |
| ref_mOTU_v3_00096 | Citrobacter sp. [ref_mOTU_v3_00096] | d__Bacteria;p__Proteobacteria;c__Gammaproteobacteria;o__Enterobacterales;f__Enterobacteriaceae;g__Citrobacter;s__Citrobacter freundii | d__Bacteria;p__Proteobacteria;c__Gammaproteobacteria;o__Enterobacterales;f__Enterobacteriaceae;g__Citrobacter;s__Citrobacter freundii |
| ref_mOTU_v3_00096 | Citrobacter sp. [ref_mOTU_v3_00096] | d__Bacteria;p__Proteobacteria;c__Gammaproteobacteria;o__Enterobacterales;f__Enterobacteriaceae;g__Citrobacter;s__Citrobacter braakii | d__Bacteria;p__Proteobacteria;c__Gammaproteobacteria;o__Enterobacterales;f__Enterobacteriaceae;g__Citrobacter;s__Citrobacter freundii |
| ref_mOTU_v3_00086 | Klebsiella michiganensis/oxytoca [ref_mOTU_v3_00086] | d__Bacteria;p__Proteobacteria;c__Gammaproteobacteria;o__Enterobacterales;f__Enterobacteriaceae;g__Klebsiella;s__Klebsiella oxytoca | d__Bacteria;p__Proteobacteria;c__Gammaproteobacteria;o__Enterobacterales;f__Enterobacteriaceae;g__Klebsiella;s__Klebsiella oxytoca |
| ref_mOTU_v3_00086 | Klebsiella michiganensis/oxytoca [ref_mOTU_v3_00086] | d__Bacteria;p__Proteobacteria;c__Gammaproteobacteria;o__Enterobacterales;f__Enterobacteriaceae;g__Klebsiella;s__Klebsiella michiganensis | d__Bacteria;p__Proteobacteria;c__Gammaproteobacteria;o__Enterobacterales;f__Enterobacteriaceae;g__Klebsiella;s__Klebsiella oxytoca |
| ref_mOTU_v3_00086 | Klebsiella michiganensis/oxytoca [ref_mOTU_v3_00086] | d__Bacteria;p__Proteobacteria;c__Gammaproteobacteria;o__Enterobacterales;f__Enterobacteriaceae;g__Klebsiella;s__Klebsiella grimontii | d__Bacteria;p__Proteobacteria;c__Gammaproteobacteria;o__Enterobacterales;f__Enterobacteriaceae;g__Klebsiella;s__Klebsiella oxytoca |
| ref_mOTU_v3_00085 | Klebsiella pneumoniae [ref_mOTU_v3_00085] | d__Bacteria;p__Proteobacteria;c__Gammaproteobacteria;o__Enterobacterales;f__Enterobacteriaceae;g__Klebsiella;s__Klebsiella pneumoniae | d__Bacteria;p__Proteobacteria;c__Gammaproteobacteria;o__Enterobacterales;f__Enterobacteriaceae;g__Klebsiella;s__Klebsiella pneumoniae |
| ref_mOTU_v3_00085 | Klebsiella pneumoniae [ref_mOTU_v3_00085] | d__Bacteria;p__Proteobacteria;c__Gammaproteobacteria;o__Enterobacterales;f__Enterobacteriaceae;g__Klebsiella;s__Klebsiella quasipneumoniae | d__Bacteria;p__Proteobacteria;c__Gammaproteobacteria;o__Enterobacterales;f__Enterobacteriaceae;g__Klebsiella;s__Klebsiella pneumoniae |
| ref_mOTU_v3_00085 | Klebsiella pneumoniae [ref_mOTU_v3_00085] | d__Bacteria;p__Proteobacteria;c__Gammaproteobacteria;o__Enterobacterales;f__Enterobacteriaceae;g__Klebsiella;s__Klebsiella variicola | d__Bacteria;p__Proteobacteria;c__Gammaproteobacteria;o__Enterobacterales;f__Enterobacteriaceae;g__Klebsiella;s__Klebsiella pneumoniae |
| ref_mOTU_v3_00077 | Enterobacter sp. [ref_mOTU_v3_00077] | d__Bacteria;p__Proteobacteria;c__Gammaproteobacteria;o__Enterobacterales;f__Enterobacteriaceae;g__Enterobacter;s__Enterobacter hormaechei_A | d__Bacteria;p__Proteobacteria;c__Gammaproteobacteria;o__Enterobacterales;f__Enterobacteriaceae;g__Enterobacter;s__Enterobacter hormaechei_A |
| ref_mOTU_v3_00077 | Enterobacter sp. [ref_mOTU_v3_00077] | d__Bacteria;p__Proteobacteria;c__Gammaproteobacteria;o__Enterobacterales;f__Enterobacteriaceae;g__Enterobacter;s__Enterobacter roggenkampii | d__Bacteria;p__Proteobacteria;c__Gammaproteobacteria;o__Enterobacterales;f__Enterobacteriaceae;g__Enterobacter;s__Enterobacter hormaechei_A |
| ref_mOTU_v3_00077 | Enterobacter sp. [ref_mOTU_v3_00077] | d__Bacteria;p__Proteobacteria;c__Gammaproteobacteria;o__Enterobacterales;f__Enterobacteriaceae;g__Enterobacter;s__Enterobacter kobei | d__Bacteria;p__Proteobacteria;c__Gammaproteobacteria;o__Enterobacterales;f__Enterobacteriaceae;g__Enterobacter;s__Enterobacter hormaechei_A |
| ref_mOTU_v3_00077 | Enterobacter sp. [ref_mOTU_v3_00077] | d__Bacteria;p__Proteobacteria;c__Gammaproteobacteria;o__Enterobacterales;f__Enterobacteriaceae;g__Enterobacter;s__Enterobacter ludwigii | d__Bacteria;p__Proteobacteria;c__Gammaproteobacteria;o__Enterobacterales;f__Enterobacteriaceae;g__Enterobacter;s__Enterobacter hormaechei_A |

**Table S5**: List of species (GTDB r207 definition) removed from specific mOTUs taxonomical annotation.

| GTDB r207 | mOTU |
| --- | --- |
| d__Bacteria;p__Bacteroidota;c__Bacteroidia;o__Bacteroidales;f__Bacteroidaceae;g__Phocaeicola;s__Phocaeicola vulgatus | ext_mOTU_v3_18052 |
| d__Bacteria;p__Bacteroidota;c__Bacteroidia;o__Bacteroidales;f__Bacteroidaceae;g__Phocaeicola;s__Phocaeicola vulgatus | ext_mOTU_v3_17856 |
| d__Bacteria;p__Bacteroidota;c__Bacteroidia;o__Bacteroidales;f__Bacteroidaceae;g__Phocaeicola;s__Phocaeicola vulgatus | ext_mOTU_v3_17779 |
| d__Bacteria;p__Cyanobacteria;c__Vampirovibrionia;o__Gastranaerophilales;f__Gastranaerophilaceae;g__CAG-196;s__CAG-196 sp002102975 | ref_mOTU_v3_02367 |
| d__Bacteria;p__Cyanobacteria;c__Vampirovibrionia;o__Gastranaerophilales;f__Gastranaerophilaceae;g__CAG-196;s__CAG-196 sp002102975 | ref_mOTU_v3_02376 |
| d__Bacteria;p__Cyanobacteria;c__Vampirovibrionia;o__Gastranaerophilales;f__Gastranaerophilaceae;g__Zag111;s__Zag111 sp002102825 | ref_mOTU_v3_02375 |
| d__Bacteria;p__Actinobacteriota;c__Coriobacteriia;o__Coriobacteriales;f__Eggerthellaceae;g__Adlercreutzia;s__Adlercreutzia celatus_A | ext_mOTU_v3_15441 |
| d__Bacteria;p__Actinobacteriota;c__Coriobacteriia;o__Coriobacteriales;f__Eggerthellaceae;g__Adlercreutzia;s__Adlercreutzia equolifaciens | ext_mOTU_v3_15443 |
| d__Bacteria;p__Bacteroidota;c__Bacteroidia;o__Bacteroidales;f__Bacteroidaceae;g__Phocaeicola;s__Phocaeicola coprocola | ext_mOTU_v3_17856 |
| d__Bacteria;p__Firmicutes_A;c__Clostridia;o__Christensenellales;f__UBA3700;g__MGBC100798;s__MGBC100798 sp910588545 | ext_mOTU_v3_18750 |

**Table S6**: Metrics of presence/absence calculated on the synthetic datasets. Values are expressed as mean ± standard deviation.

|  | Meteor2 | Meteor2_fast | MetaPhlAn4 | sylph | mOTUs3 | KMCP | SingleM | Kraken2 | ganon2 |
| --- | --- | --- | --- | --- | --- | --- | --- | --- | --- |
| Sensitivity (%) | 99.9 ± 0.2 | 98.9 ± 1.2 | 94.4 ± 2.7 | 93.2 ± 2.8 | 94.5 ± 2.4 | 99.3 ± 0.7 | 45.9 ± 6.7 | 99.3 ± 0.6 | 94.5 ± 2.7 |
| Specificity (%) | 98.8 ± 0.9 | 96.2 ± 1.9 | 99.9 ± 0.2 | 99.2 ± 0.7 | 94.8 ± 2.7 | 63 ± 11.3 | 94.2 ± 4 | 26.4 ± 5.7 | 55.5 ± 12.2 |
| True Positives | 162.7 ± 39.2 | 161 ± 38.9 | 153.9 ± 38.3 | 151.9 ± 37.4 | 151.6 ± 35.8 | 161.7 ± 39.1 | 74.2 ± 19 | 161.6 ± 38.9 | 153.9 ± 37.8 |
| False Positives | 1.9 ± 1.6 | 5.9 ± 2.5 | 0.1 ± 0.3 | 1.1 ± 1.1 | 8.4 ± 4.9 | 99.9 ± 45.4 | 4.7 ± 3.6 | 460.8 ± 115.1 | 128.1 ± 48.9 |
| False Negatives | 0.1 ± 0.4 | 1.8 ± 1.7 | 8.9 ± 4.3 | 10.9 ± 4.7 | 9.1 ± 4.9 | 1.1 ± 1.1 | 88.6 ± 25.4 | 1.2 ± 1 | 8.9 ± 4.6 |

**Table S7**: Performance in terms of time and memory usage of the different tools on the synthetic datasets. Values are expressed as mean ± standard deviation.

| Level | Tool | Ecosystem | Mean wall time (min) | Max memory (GB) |
| --- | --- | --- | --- | --- |
| taxonomy+functional | Meteor2 | Human gut | 39.8 ± 2.7 | 51.5 ± 0.6 |
| taxonomy+functional |  | Mouse gut | 20.4 ± 1.8 | 32.8 ± 0.5 |
| taxonomy | Meteor2_fast | Human gut | 2.1 ± 0.1 | 3.6 ± 0.1 |
| taxonomy |  | Mouse gut | 2.1 ± 0.1 | 3.1 ± 0.2 |
| taxonomy | MetaPhlAn4 | Human gut | 11.8 ± 0.2 | 17.9 ± 0 |
| taxonomy |  | Mouse gut | 11.6 ± 0.2 | 18 ± 0 |
| taxonomy | sylph | Human gut | 1.5 ± 0.3 | 9.8 ± 0.1 |
| taxonomy |  | Mouse gut | 1.5 ± 0.3 | 9.8 ± 0.1 |
| taxonomy | mOTUs3 | Human gut | 9 ± 0.6 | 4.6 ± 0 |
| taxonomy |  | Mouse gut | 7.7 ± 0.3 | 4.6 ± 0 |
| taxonomy | KMCP | Human gut | 587.8 ± 4.3 | 84.3 ± 0.5 |
| taxonomy |  | Mouse gut | 579.7 ± 4.2 | 83.5 ± 0.7 |
| taxonomy | SingleM | Human gut | 9.9 ± 0.5 | 2.7 ± 0.1 |
| taxonomy |  | Mouse gut | 9.9 ± 0.5 | 2.6 ± 0.1 |
| taxonomy | Kraken2 | Human gut | 9.8 ± 2.7 | 301.7 ± 0.1 |
| taxonomy |  | Mouse gut | 18.2 ± 0.4 | 301.7 ± 0.1 |
| taxonomy | ganon2 | Human gut | 26.2 ± 3.7 | 172.9 ± 0 |
| taxonomy |  | Mouse gut | 24.9 ± 1.9 | 172.9 ± 0 |
| functional | HUMAnN3 | Human gut | 127.6 ± 13 | 17.9 ± 0 |
| functional |  | Mouse gut | 162.9 ± 27.5 | 18.5 ± 0.4 |
| strain | Meteor2 | Human gut | 41.1 ± 9.2 | 26.9 ± 0.4 |
| strain |  | Mouse gut | 26.8 ± 5.9 | 14.5 ± 0.2 |
| strain | Meteor2_fast | Human gut | 12.9 ± 2.4 | 3.5 ± 0 |
| strain |  | Mouse gut | 10.5 ± 2.1 | 2.8 ± 0 |
| strain | StrainPhlAn4 | Human gut | 13.5 ± 0.4 | 4.9 ± 0 |
| strain |  | Mouse gut | 13.4 ± 0.3 | 4.9 ± 0 |
| strain | inStrain | Human gut | 76.8 ± 6.7 | 63.6 ± 0 |
| strain |  | Mouse gut | 50.8 ± 6.7 | 26.4 ± 3 |

**Table S8**: AUC of tools’ ability to discriminate between identical genomes (100% ANI) and distinct genomes (< 100% ANI).

| ecosystem | tool | auc |
| --- | --- | --- |
| human | Meteor2 (mixed) | 0.99 |
| human | Meteor2 (dominant) | 0.98 |
| human | Meteor2 fast (mixed) | 0.99 |
| human | Meteor2 fast (dominant) | 0.99 |
| human | StrainPhlAn4 | 0.99 |
| human | inStrain (popANI) | 0.99 |
| human | inStrain (conANI) | 0.96 |
| mouse | Meteor2 (mixed) | 0.94 |
| mouse | Meteor2 (dominant) | 0.93 |
| mouse | Meteor2 fast (mixed) | 0.94 |
| mouse | Meteor2 fast (dominant) | 0.92 |
| mouse | StrainPhlAn4 | 0.94 |
| mouse | inStrain (popANI) | 0.98 |
| mouse | inStrain (conANI) | 0.94 |

Supplementary Figures

**Figure** S**1:** Pairwise ANI of genomes used to generate simulated samples.


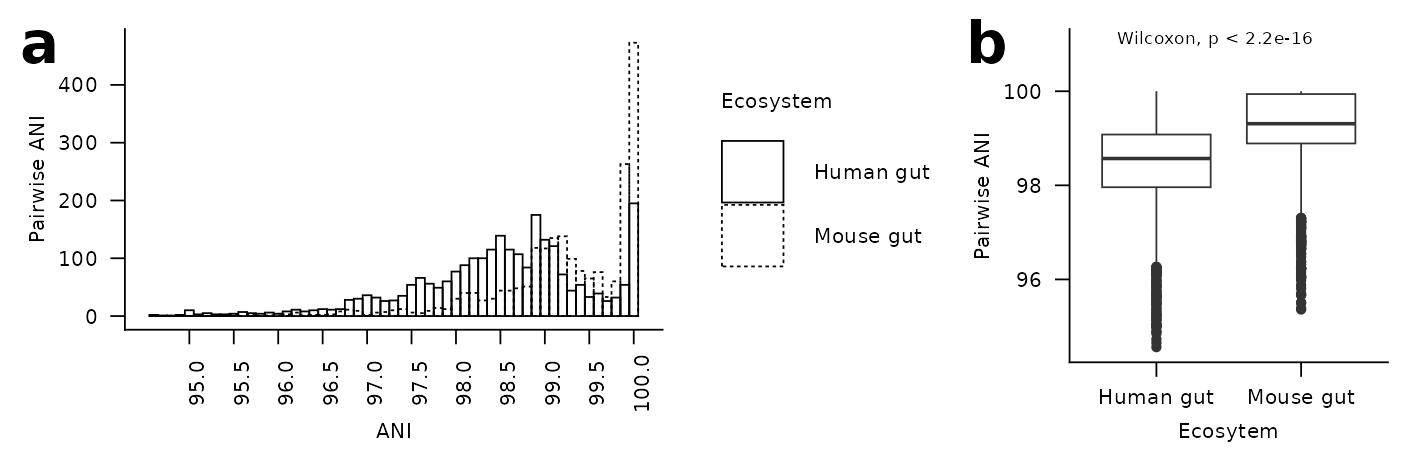


**a.** Histogram and **b.** boxplot of %ANI values computed between genomes used to generate simulated samples, in the human gut and the mouse gut ecosystem. P-value associated with Wilcoxon test is shown.

**Figure S2**: Distribution of the depth coverage of true positive and false negative species for MetaPhlAn4 and sylph.


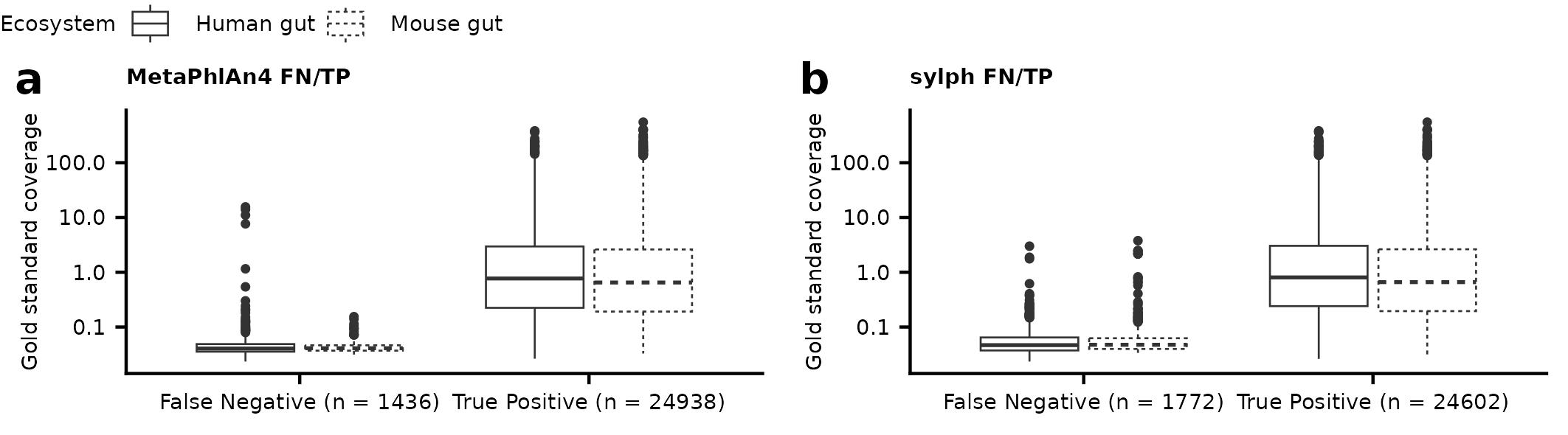


**a**, **b**, The results showed that 1436 and 1772 species in total, respectively, were not detected by these tools, despite being present in the metagenomic samples. These false negative species detections were characterized by a low relative abundance, with majority of them having a coverage below 1X.

**Figure S3**: Meteor2 accurately profiles high- and low-abundance species from synthetic metagenomes.


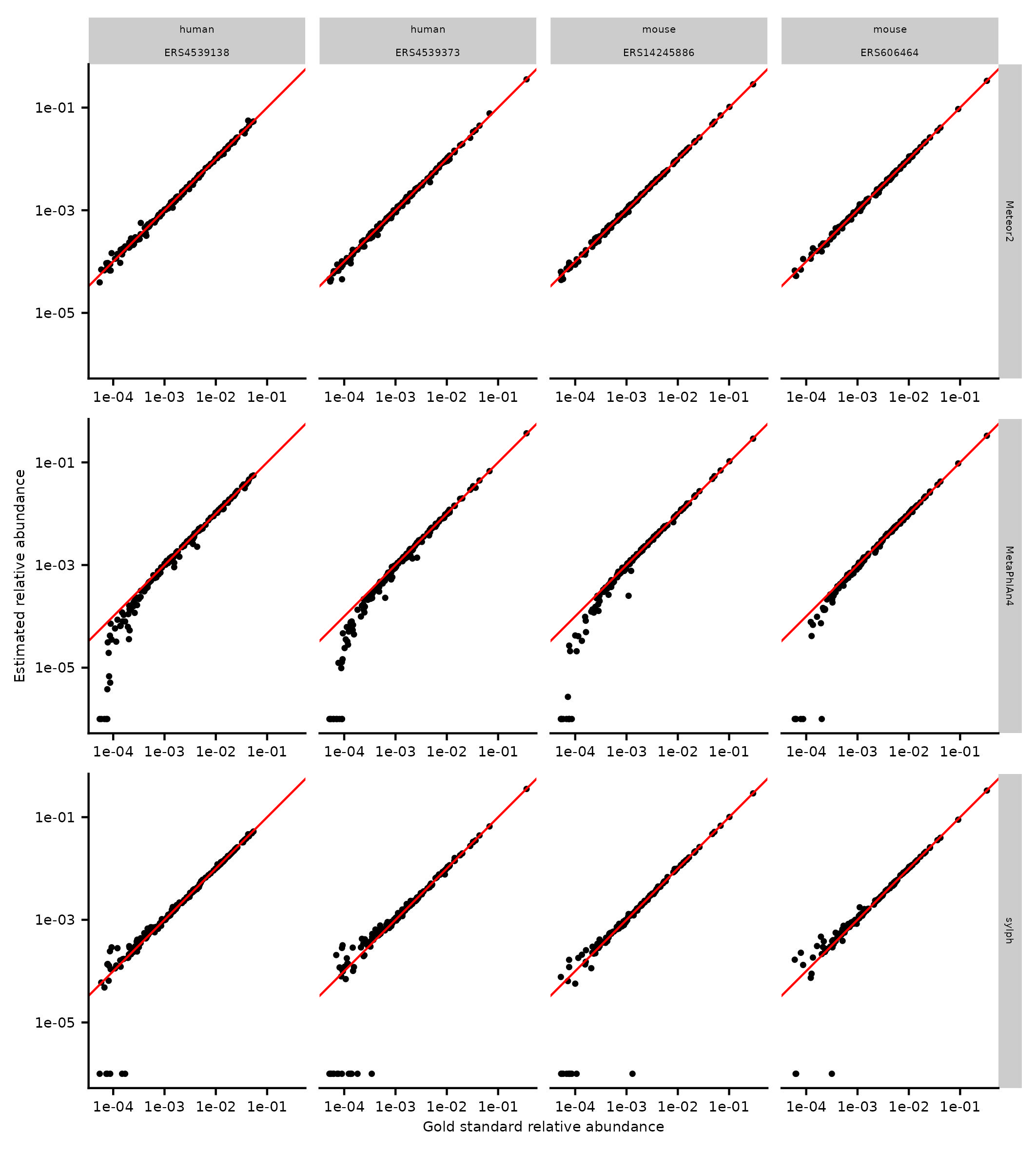


Taxonomic profiles of four synthetic metagenomic samples from human and mouse gut ecosystems, generated using MetaPhlAn4, Meteor2, and sylph. The estimated abundance is compared to the gold standard, with ideal performance indicated by a correlation along the line y=x (red line).

**Figure S4**: Taxonomic profiling performance of Meteor2 in comparison to MetaPhlAn4 and sylph for low sequencing depths.


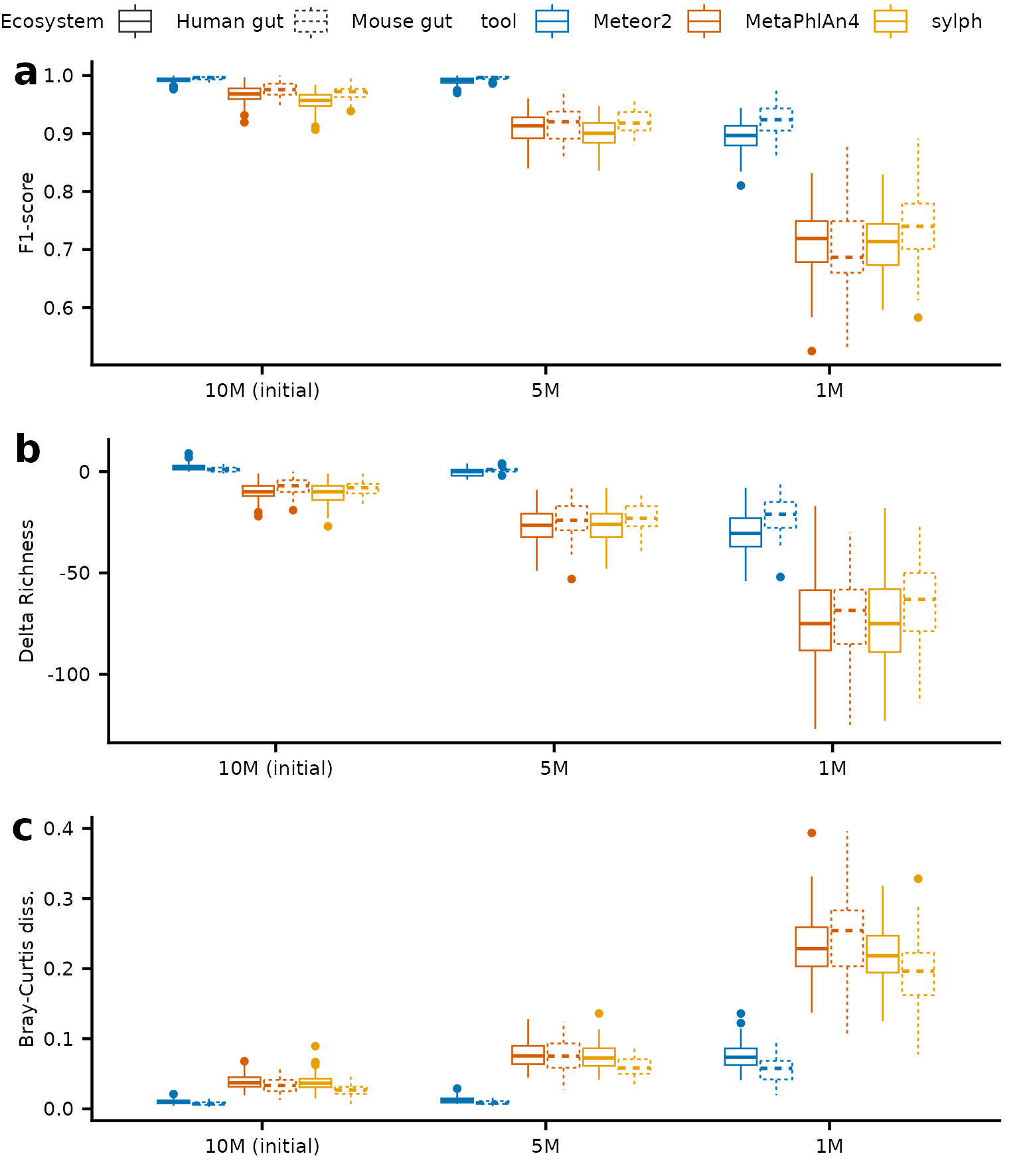


**a**, Taxonomic profiling performance according to F1-score of Meteor2, MetaPhlAn4, and sylph at low sequencing depths (5 or 1 million paired read depth) compared to original depth (10 million paired read depth) for synthetic dataset. **b**, Richness delta, corresponding to the difference between gold standard richness and tools’ estimated richness. **c**, Bray-Curtis dissimilarity values computed between the log10-transformed estimated profiles and the abundances in the gold standard.

**Figure S5**: Functional profiling performance of Meteor2 in comparison to HUMAnN3


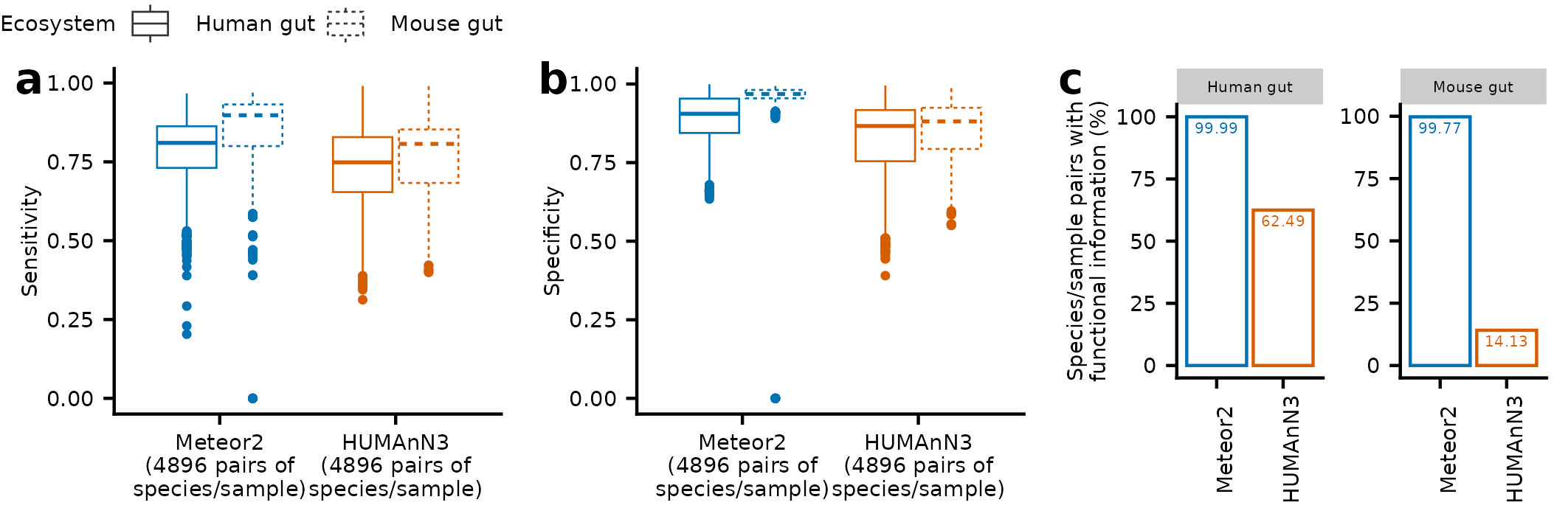


Evaluation of (**a**) sensitivity and (**b**) specificity for functional content detection for Meteor2 and HUMAnN3 for human and mouse synthetic metagenome. (**c**) Percentage of species/sample pairs for which functional information could be retrieved, among all species/sample pairs with gold standard coverage above 1X.

**Figure S6**: Relationship between pairwise ANI and mutation rate distributions.


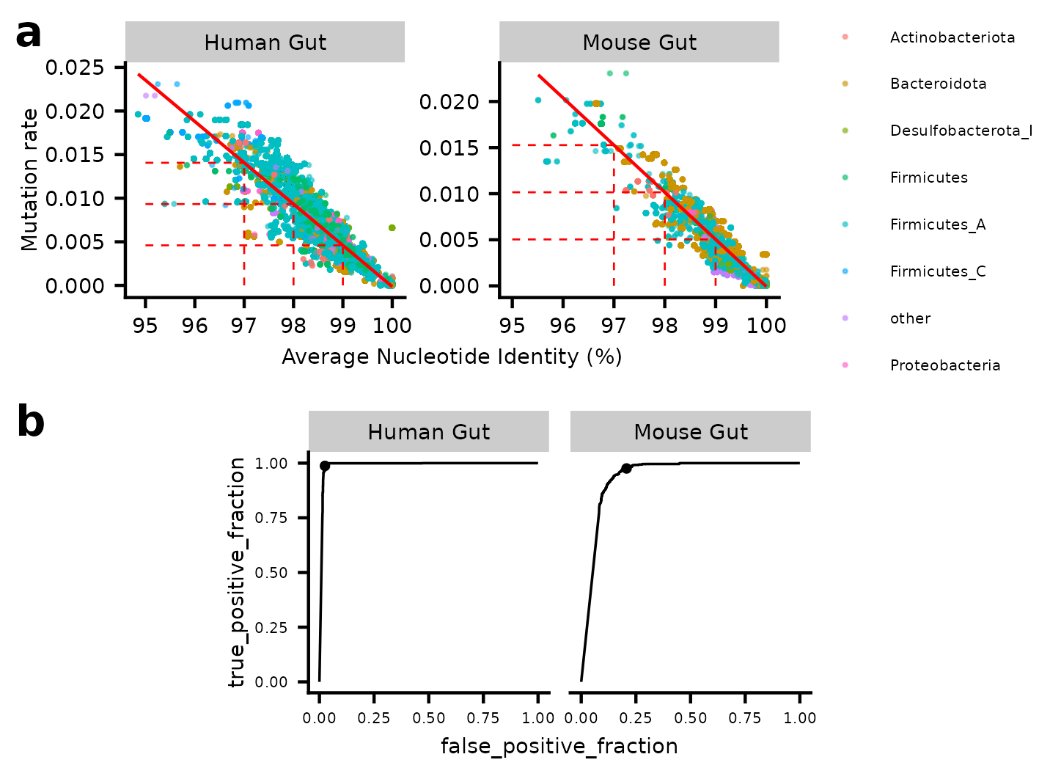


**a**, Relationship between the gold standard ANI and the mutation rates estimated by Meteor2 (considering all alleles - mixed configuration). The solid red line represents the linear regression between ANI and the mutation rate, while the dashed red segments indicate the estimated mutation rate corresponding to 97%, 98% and 99% ANI thresholds. The points are colored according to the phylum of the MSP, with phyla having fewer than 1,000 points categorized as “other”. **b**, The Receiver Operating Characteristic (ROC) curve evaluates the ability of Meteor2-estimated mutation rate to distinguish between identical genomes (ANI = 100%) and non-identical genomes (ANI < 100%), with the point on the curve indicating the chosen cut-off at 1e-04.

**Figure S7:** Strain discrimination performance for genomes from different subspecies


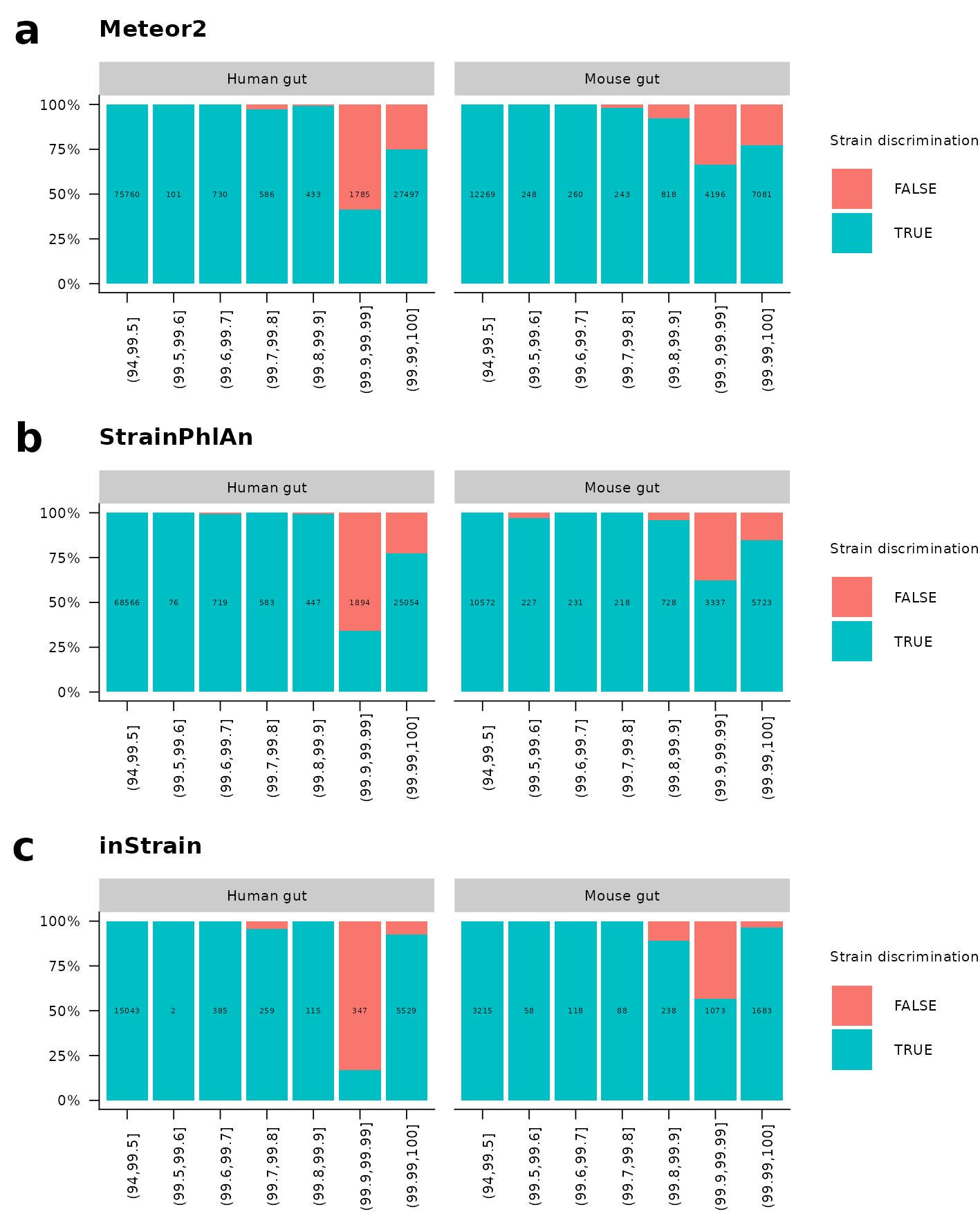


Pairs of strains are grouped by their ANI (x-axis) and by the ability of (a) Meteor2, (b) StrainPhlAn or (c) inStrain to correctly classify them as identical or different (bar color). Strains are considered identical when the tool’s measure meets a stringent threshold (inferior to 2.99e-12 for Meteor2, equal to 0 for StrainPhlAn or superior to 99.995 for inStrain). The category (99.99,100] corresponds to truly identical strains. Numbers on each bar indicate the number of pairs in each ANI category.

**Figure S8**: Meteor2 provides insights into a real FMT dataset at diversity, taxonomic, functional and strain level.


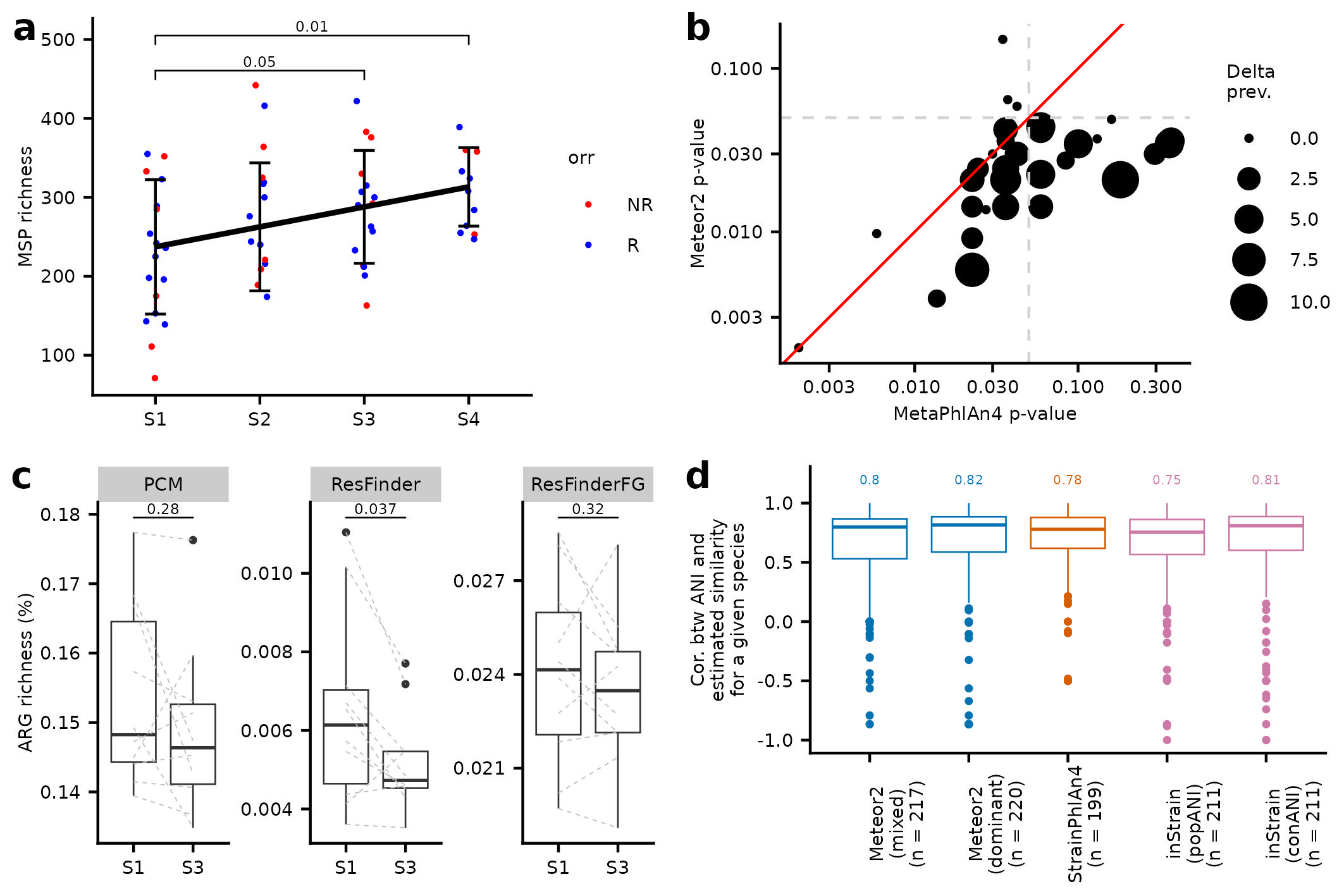


**a**, Alpha-diversity, estimated by MSP richness in gut microbiota of recipients across different timepoints. Wilcoxon tests p-values are displayed. **b**, P-values for species differentially abundant between S1 and S3 (in responders only), computed via Wilcoxon signed-rank tests using either MetaPhlAn4 or Meteor2. The correspondence between MSP (Meteor2) and SGB (MetaPhlan4) is based on GTDB r207. **c**, Distribution of ARG richness, expressed as a percentage of total gene richness, comparing S1 and S3 (responders only). Wilcoxon signed-rank tests p-values are displayed. ARG richness is determined by gene functional annotation using three complementary ARG databases integrated into Meteor2. **d**, Distribution of species-wise Spearman’s rho between ANI (estimated from MAGs built for each sample) and inferred similarity metrics (1 - mutation rate for Meteor2 and StrainPhlAn4, ANI for inStrain).

**Figure S9**: Engraftment rate computed on real FMT dataset using ANI or mutation rate provided by different tools and different configurations.


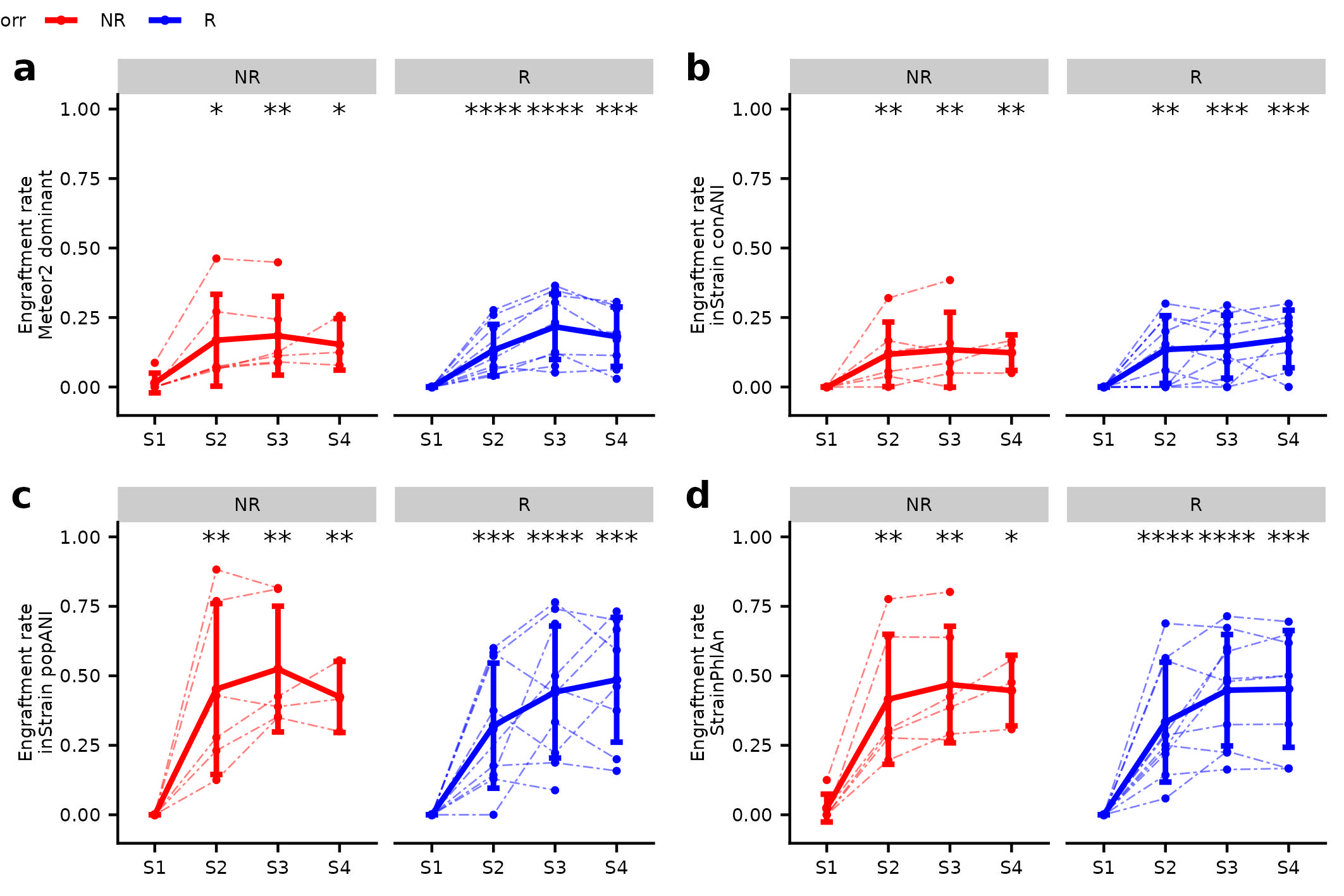


Engraftment rate in responder (R) and non-responders (NR) are shown at all timepoints, for **a**, Meteor2 dominant configuration, **b**, inStrain conANI, **c**, inStrain popANI, **d**, StrainPhlAn4. Each point represents an individual sample, with samples from the same individual connected by dashed lines. The solid line indicates the mean engraftment rate across all samples at each time point, and error bars represent the standard deviation. P-values from Wilcoxon tests (relative to S1) are displayed. * indicates 0.01 < p-value ≤ 0.05, **: 0.001 < p-value ≤ 0.01, ***: 0.0001 < p-value ≤ 0.001, ****: p-value ≤ 0.0001.

**Figure S10**: TFSP integration: clinical, taxonomic, functional and strain information on *Bacteroides stercoris* (msp_0032).


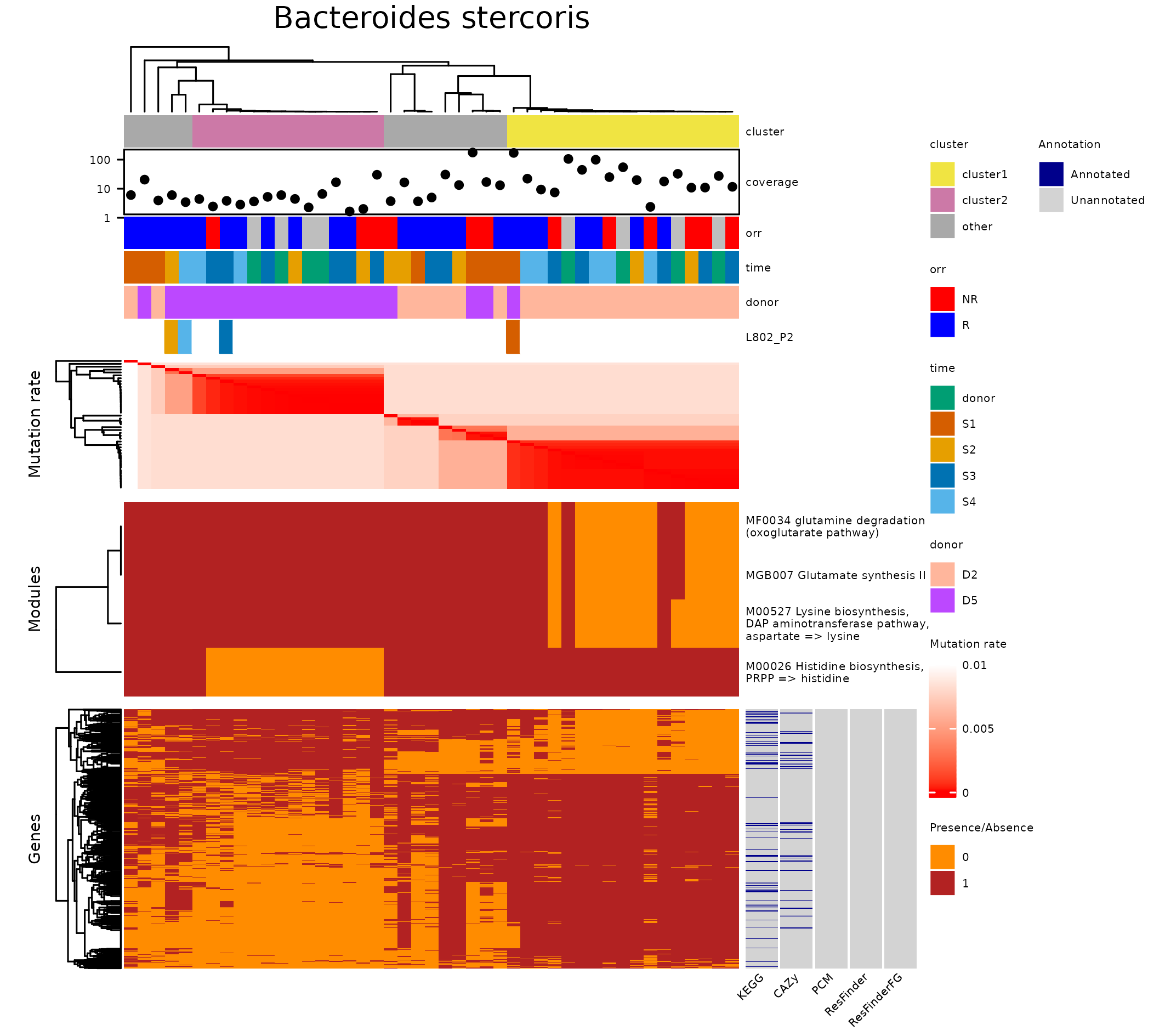


A total of 45 samples were available for strain analysis. Hierarchical clustering of the mutation rate matrix was performed using complete linkage, and clusters were defined by cutting the dendrogram at h = 0.005 (corresponding to 99% ANI). The two largest clusters (n = 17 and n = 14) are highlighted in yellow and pink, respectively, while the remaining samples are grouped under 'other' (grey). For each sample, MSP coverage is shown along with the objective response rate (ORR) status (responder or non-responder), timepoint, and donor information. Donor samples are marked with ORR = NA (grey) and labelled as ‘donor’ timepoints (green). Samples from individual L802_P2 are highlighted by their respective timepoints. Below the mutation rate matrix, presence/absence matrices of functional modules and accessory genes of the MSP are displayed, contrasting the yellow and pink clusters (Chi-squared test, p < 0.05). On the right, annotation of the contrasted genes is provided from all available databases.

Supplementary Information

**Le French Gut Consortium Members**

Mathieu Almeida^1^, Anne-Sophie Alvarez^1^, Monique Axelos^2^, Mourad Benallaoua^3^, Robert Benamouzig^3^, Oana Bernard^4^, Sylvie Binda^5^, Hervé M. Blottière^1,6^, Elise Borezée^1,7^, Alexandre Cavezza^1^, Pierre Cressard^8^, Chloe Connan^1^, Anne-Marie Davila-Gay^9^, Christophe d’Enfert^10^, Joël Doré^1,7^, Assia Dreux-Zigha^11^, Erik Eckhardt^12^, S Dusko Ehrlich^1^, Etienne Formstecher^13^, Sebastien Fromentin^1^, Amine Ghozlane^14^, Florence Haimet^1,15^, Maina Houssaye^16^, Milan Lazarevic^17^, Sophie Legrain^18^, Françoise Levacon^19^, Christian Morabito^1^, Pedro H. Oliveira^20^, Raish Oozeer^21^, Florian Plaza-Onate^1^, Nicolas Pons^1^, Benoît Quinquis^1^, Karine Roget^22^, Florence Thirion^1^, Karine Valeille^1^, Patrick Veiga^1,7^

^1^ Université Paris-Saclay, INRAE, MetaGenoPolis (MGP), 78350 Jouy-en-Josas, France

^2^INRAE, Paris, France.

^3^ Department of Gastroenterology, Avicenne Hospital, Assistance Publique-Hôpitaux de Paris, Université de Paris, Bobigny, France.

^4^Biocodex, Compiegne, France.

^5^Lallemand Health Solutions, Toulouse, France.

^6^ Nantes Université, INRAE, UMR 1280, PhAN, F-44000 Nantes, France

^7^ Université Paris-Saclay, INRAE, AgroParisTech, Micalis Institute, 78350 Jouy-en-Josas, France

^8^ Nahibu, Rennes, France.

^9^ Université Paris-Saclay, AgroParisTech, INRAE, UMR PNCA, 91120, Palaiseau, France.

^10^Institut Pasteur, Direction Générale Adjointe Scientifique, F-75015 Paris, France.

^11^Greentech, Saint-Beauzire, France.

^12^Adare Biome, Houdan, France.

^13^GMT Science, Rouen, France.

^14^Institut Pasteur, Université Paris Cité, Bioinformatics and Biostatistics Hub, F-75015 Paris, France.

^15^ Mica division, 78350 Jouy-en-Josas, France

^16^INSERM, Paris, France.

^17^APHP, Paris, France.

^18^Gnosis by Lesaffre, Marcq-en-Baroeul, France

^19^Biofortis, Saint-Herblain, France.

^20^Génomique Métabolique, Genoscope, Institut François Jacob, CEA, CNRS, Université Evry, Université Paris-Saclay, Evry, France.

^21^Danone Nutricia Research, Gif-sur-Yvette, France.

^22^Nextbiome Therapeutics, Clermont-Ferrand, France.
